# Smart H_2_S‐Triggered/Therapeutic System (SHTS)‐Based Nanomedicine

**DOI:** 10.1002/advs.201901724

**Published:** 2019-10-14

**Authors:** Weiyu Chen, Dalong Ni, Zachary T. Rosenkrans, Tianye Cao, Weibo Cai

**Affiliations:** ^1^ Departments of Radiology and Medical Physics University of Wisconsin‐Madison Madison WI 53705 USA; ^2^ Department of Pharmaceutical Sciences University of Wisconsin‐Madison Madison WI 53705 USA

**Keywords:** gas delivery, H_2_S‐specific detection, H_2_S‐triggered therapy, hydrogen sulfide, novel nanoplatforms

## Abstract

Hydrogen sulfide (H_2_S) is of vital importance in several biological and physical processes. The significance of H_2_S‐specific detection and monitoring is emphasized by its elevated levels in various diseases such as cancer. Nanotechnology enhances the performance of chemical sensing nanoprobes due to the enhanced efficiency and sensitivity. Recently, extensive research efforts have been dedicated to developing novel smart H_2_S‐triggered/therapeutic system (SHTS) nanoplatforms for H_2_S‐activated sensing, imaging, and therapy. Herein, the latest SHTS‐based nanomaterials are summarized and discussed in detail. In addition, therapeutic strategies mediated by endogenous H_2_S as a trigger or exogenous H_2_S delivery are also included. A comprehensive understanding of the current status of SHTS‐based strategies will greatly facilitate innovation in this field. Lastly, the challenges and key issues related to the design and development of SHTS‐based nanomaterials (e.g., morphology, surface modification, therapeutic strategies, appropriate application, and selection of nanomaterials) are outlined.

## Graphical abstract

H_2_S‐specific nanoagents are of vital importance in detecting endogenous and exogenous H_2_S gas. The recent developments of these smart H_2_S‐triggered/therapeutic systems (SHTSs) are summarized. Additionally, the challenge faced, and the principals involved, are further discussed for future H_2_S‐nanoprobe designs.

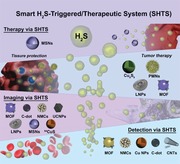

## 1 Introduction

Hydrogen sulfide (H_2_S) is a highly toxic gas known for its causticity, flammability and distinct odor of rotten eggs.^1^, ^2^, ^3^ However, endogenous H_2_S is the third major gasotransmitter in addition to carbon monoxide (CO) and nitric oxide (NO).^4^, ^5^, ^6^ The misregulation of this signaling molecule is associated with numerous diseases, such as Alzheimer's disease, diabetes, and cancer.^7^, ^8^ Since H_2_S has such a crucial role, an effective H_2_S detection method would facilitate the understanding of the implicated diseases permit early diagnosis. Currently, the most‐used techniques include high‐pressure liquid/gas chromatography (HPLC/GC) have shown significant sensitivity. However, the high cost and tediously processing time severely restrict their practical application in detecting H_2_S in biological samples, especially for real‐time measurements. In comparison, novel small molecules ranging from colorimetric and fluorescent probes have demonstrated substantial advantages for dynamic and in situ H_2_S sensing/imaging via various chemical strategies.^9^ Several fluorescent probes, such as sulfidefluor‐1/2 (SF‐1/2) and hydrogen sulfide imaging probe‐1 (HSip‐1) present desirable selectivity and can “turn on” an H_2_S‐activated fluorescent signal for H_2_S detection (e.g., living cell imaging), with limits of detection (LOD) (all the abbreviations could be found in **Table** **1**) reported around 5 × 10^−6^–10 × 10^−6^ m.^10^, ^11^, ^12^ While well‐designed small molecule probes have been applied for H_2_S‐selective detection in live cells and in vivo imaging,^13^, ^14^ they still present issues such as the relatively low sensitivity and selectivity, poor water solubility, weak fluorescent intensity,^13^ and poor circulation (e.g., the accumulation in liver) that must be overcome.^14^

**Table 1:** Full names and the corresponding abbreviations

Full name	Abbreviation	Full name	Abbreviation
3‐mercaptopyruvate sulfotransferase	3‐MST	Myocardial infarction	MI
Aerosol‐assisted chemical vapor deposition	AACVD	Metal‐organic frameworks	MOF
Alzheimer's Disease	AD	11‐mercaptoundecanoic acid	MUA
Anethole dithiolethione	ADT	Mesoporous silica nanoparticles	MSNs
Aggregation‐induced emission	AEI	Near Infrared	NIR
Anethole dithiolethione (ADT)‐loaded magnetic nanoliposome	AMLs	Noble metal clusters	NMCs
Amino‐oxyacetic acid	AOAA	Photoacoustic	PA
Adenosine triphosphate	ATP	Positron emission tomography	PET
Carbon nanodots	C‐dot	Photodynamic therapy	PDT
Cystathionine β‐synthase	CBS	Polymeric nanoparticles	PMNs
Carbon nanotubes	CNTs	Plasmonic nanoparticles	PNPs
Cystathionine γ‐lyase	CSE	Polystyrene sulfonate	PSS
Diallyl sulfide	DATS	Photothermal therapy	PTT
Functional graphene sheets	FGS	Reactive oxygen species	ROS
Ischemia/reperfusion	I/R	S‐adenosyl‐l‐methionine	SAM
Inner filter effect	IFE	Smart H_2_S‐triggered/therapeutic system	SHTS
Intercellular adhesion molecule‐1	ICAM‐1	Tris(2‐chloroisopropyl)phosphate	TCPP
Limits of detection	LOD	3,3′,5,5′‐tetramethylbenzidine	TMB
Liposome nanoparticles	LNPs	1‐(10‐mercaptodecyl)‐5‐methylpyrimidine‐2,4‐dione	TSH
Luminescence/Förster resonance energy transfer	LRET/FRET	Triphenyltetrazolium chloride	TTC
Longitudinal surface plasmon resonance's	LSPR	Upconverting nanoparticles	UCNPs
Vascular cell adhesion molecule‐1	VCAM‐1	Upconversion luminescence	UCL

During the recent two decades, nanomaterials have drawn substantial global attention.^15^, ^16^, ^17^, ^18^, ^19^ Due to their desirable physiochemical features (e.g., high biocomparability and stability, large specific surface area, excellent loading efficiency, variable modification, etc.),^20^, ^21^, ^22^ nanomaterials have been widely employed in various biomedical applications in drug delivery, vaccination, imaging, and therapy.^23^, ^24^, ^25^, ^26^, ^27^, ^28^, ^29^, ^30^, ^31^, ^32^, ^33^ With careful design, nanoplatforms can exceed small molecule probes as ideal sensing agents for rapid, selective, and efficient H_2_S detection.^34^, ^35^, ^36^, ^37^ Recently, novel nanoprobes have been developed for H_2_S sensing, which efficiently detect and image hydrogen sulfide via 1) chemical features of the loaded smart fluorophore (e.g., Aziede reduction, metal precipitation and nucleophilic attack),^38^, ^39^, ^40^ 2) change of localized surface plasmon resonance's (LSPR),^41^, ^42^, ^43^ 3) variation of absorbance (colorimetric assay),^44^ 4) surface metal precipitation,^45^, ^46^, ^47^ 5) change of luminescence/Förster resonance energy transfer (LRET/FRET),^48^, ^49^, ^50^ or 6) electrochemical reaction.^51^, ^52^, ^53^ More importantly, a series of therapeutic strategies (e.g., photothermal therapy and photodynamic therapy, etc.) could be intelligently triggered by the activation of endogenous H_2_S from the targeting area.^54^, ^55^ Additionally, exogenous H_2_S delivery has been successfully achieved via nanoplatforms, which can induce H_2_S‐mediated gas therapy (via physical damage) or tissue protection (e.g., the heart I/R injury) within the disease regions.^56^, ^57^ These multifunctional nanoplatforms may generate novel treatments available for various H_2_S‐related diseases.

**Figure 1:**
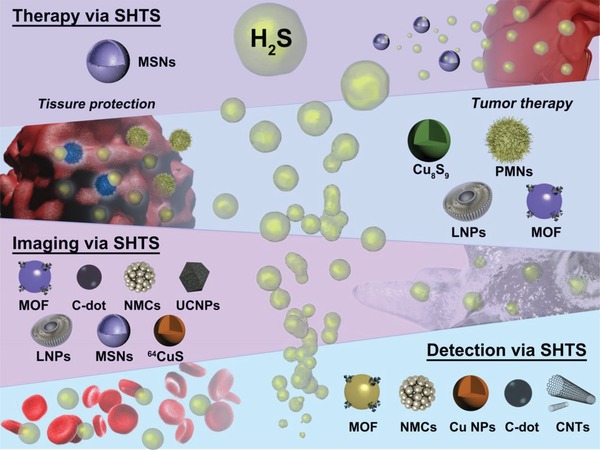
The H_2_S‐specific detection, imaging, and therapy mediated by the smart H_2_S‐triggered/therapeutic system (SHTS).

As such a promising field, smart H_2_S‐triggered/therapeutic system (SHTS)‐based nanomedicine is expected to significantly accelerate the development of disease diagnosis and therapeutic strategy by enhancing accuracy and efficiency. Given the vital role of hydrogen sulfide in biological processes and advantages of nanotechnology, we provide an overview of recent progress in H_2_S detection, imaging and related disease therapy via SHTS‐based nanomedicine (**Figure** **1**). Within this review, various nanoagents such as noble metal nanomaterials, metal‐organic framework, copper‐based nanomaterials, and carbon nanodot for H_2_S sensing, different imaging (including fluorescence, localized surface plasmon resonance, upconversion luminescence, near‐infrared, photoacoustic and positron emission tomography imaging) and therapeutic strategies (e.g., the endogenous H_2_S‐triggered therapy or exogenous H_2_S delivery) are summarized. As such, we aim to highlight these powerful nanoprobes in this emerging field and offer an overview for the development of next‐generation of SHTS‐based nanomedicine.

## 2 Roles of H_2_S in Biological Systems

Endogenous H_2_S is mainly produced from cysteine by three enzymes: 3‐mercaptopyruvate sulfotransferase (3‐MST), cystathionine β‐synthase (CBS), and cystathionine γ‐lyase (CSE).^58^, ^59^, ^60^, ^61^ The H_2_S generated is a vital gas transmitter that affects various biological and physical functions within the body, ranging from antiinflammation to regulation of neuronal transmission.^62^, ^63^, ^64^, ^65^ For instance, it has been reported that H_2_S donors promote the production of ATP and electron transport in mitochondrial.^66^ Furthermore, H_2_S is able to protect the cell by attenuating apoptosis.

Thus, it has been widely applied as a novel reagent for preserving organs from ischemia‐reperfusion injury during various surgeries and organ transplantations.^67^, ^68^, ^69^ Also, the increased secretion of endogenously H_2_S is strongly associated with the progress of tumor.^4^, ^70^

Notably, the H_2_S generating enzymic system including 3‐MST, CBS, and CSE have been widely identified in many cancer types.^4^, ^71^ The overexpression of CBS has been particularly reported within various colon and ovarian cancers,^72^, ^73^ indicating the significant role of H_2_S in promoting tumor development. The hydrogen sulfide derived from cancer cells also promotes tumor growth and proliferation by acting as an autocrine and paracrine factor.^72^ After introducing a CBS inhibitor, the growth of colon cancer could be greatly attenuated by efficiently reducing H_2_S generation and inhibiting peritumor angiogenesis.^61^ However, the fast catabolism and regulation of this toxic gas show a great challenge for real‐time detection within the tissues.^74^ As one of the most dangerous gases, the concentration of H_2_S within the air needs to be monitored as well. While this toxic gas easily noted because of its rotten‐egg smell, the exposure to H_2_S can cause a serial of symptoms including lung irritations (≤20 ppm), damage of eye (300–500 ppm), unconsciousness, or even death (≥700 ppm).^75^ Therefore, successful detection/imaging of hydrogen sulfide would be immensely valuable for disease diagnosis and treatment, as well as risk management.

## 3 H_2_S Detection with SHTS‐Based Nanomedicine

To monitor H_2_S in solutions and air, various nanomaterials have been developed as novel sensors, including noble metal nanoparticles (e.g., Au, Ag, and Au/Ag alloy), metal‐organic frameworks (MOF), copper nanomaterials, carbon nanodots, among others (e.g., ruthenium nanoparticles, etc.). In this section, a series of SHTS‐based nanosensors will be summarized (**Table** **2**).

**Table 2:** The nanosized materials as SHTS for H_2_S detection

Material^a)^	Nanoparticle	Size [nm]	Mechanism	Assay	Sample Phase	LOD	Ref.
Au	Au NRs	≈60	Aggregation	Colorimetry (A730)	Solution	24 × 10^−6^ m	76
	AEAuNPs	13.3 ± 1.6	Aggregation	Colorimetry (A520/720)	Solution	20 × 10^−6^ m	44
	GSH‐AuNP	13	Aggregation	Colorimetry (A700/520)	Solution	3 × 10^−6^ m	77
	BSA‐AuNCs‐HSIP‐1	≈1	Aggregation	Metal precipitation (I519/I632)	Solution	0.73 × 10^−6^ m	78
	TSH‐MUA‐ AuNDs	1.9 ± 0.3	Antiaggregation	Fluorescence (Em510)	Solution	0.5 × 10^−6^ m	79
	Cu@Au NPs	N/A	Competitive binding	Colorimetry (A650/520)	Solution	0.3 × 10^−6^ m	80
	AuS/Au NPs	N/A	Reduction	Colorimetry (A414)	Solution	0.28 × 10^−6^ m	81
	FSN‐AuNRs	30.8 ± 2 × 12.5 ± 1	Aggregation	Colorimetry (A518/A648)	Solution, Serum	0.2 × 10^−6^ m	82
	Au NPs	8.1 ± 1.1	Catalyst	Colorimetry (A652)	Solution	80 × 10^−9^ m	83
	Au NPs	13	Antiaggregation	Colorimetry (A520)	Bubble gas	30 × 10^−9^ m	84
	Au@TPt‐NCs	17.1	Catalyst	Colorimetry (A650)	Solution/Evaporated gas	7.5 × 10^−9^ m	85
Au/Ag	Core–shell Au@Ag NCs	≈1.8	Quench of the fluorescence	Fluorescence (Em650)	Solution	0.31 × 10^−6^ m	86
	DNA‐Au/Ag NCs	1.6	Quench of the fluorescence	Fluorescence (Em440)	Solution	0.83 × 10^−9^ m	87
Ag	C314‐Ag NPs	6–14	Reduction	Fluorescence (Em493)	Solution	≈60 ppb	88
	Chit‐AgNPs	9 ± 2.5	LSPR change	Colorimetry (A404)	Solution	0.35 × 10^−6^ m	42
	PPF cage‐AgNPs	6–8.4	LSPR change	Colorimetry (A400)	Solution	0.2 × 10^−6^ m	43
MOF	Eu^3+^/Cu^2+^@UiO‐66‐(COOH)_2_	80–100	Metal precipitation	Fluorescence (I615/I393)	Solution	5.45 × 10^−6^ m	46
	Tb^3+^@Cu‐MOF	N/A	Metal precipitation	Fluorescence (I544/I390)	Solution	1.2 × 10^−6^ m	89
	[[EuCu(pydc)_2_(ox)_0.5_(H_2_O)_3_·1.5H_2_O]_2n_	N/A	Catalyst	Fluorescence (Em615)	Solution, Serum	130 × 10^−9^ m	90
	Al‐MIL‐53‐NO_2_ MMMs	60–80	Reduction	Fluorescence (Em466)	Solution	92.31 × 10^−9^ m	39
	Zr(TBAPy)_5_(TCPP)	≈100	Reduction	Fluorescence (≈Em440)	Solution	1 ppb	91
	Cu‐SWCNTs	N/A	Reduction	Electrochemistry	Solution	5 ppm	92
Cu	p‐CuO/*n*‐SnO_2_ NWs	200 (CuO)	Breakup of pn junction	Electrochemistry	Gas	1 ppm	93
	Quasi‐2D Cu_2_O/SnO_2_	N/A	Reduction	Electrochemistry	Gas	0.5 ppm	94
	Cu_2_O‐WO_3_ NDs	2–3 (Cu_2_O)	Reduction	Electrochemistry	Gas	300 ppb	53
	PSS‐PAE‐Cu NCs	173	Aggregation	Fluorescence (Em665)	Solution	650 × 10^−9^ m	95
	Cu_2_O–FGS	3 (Cu_2_O)	Reduction	Electrochemistry	Gas	5 ppb	52
	Cysteine‐Cu NCs	2.5	Aggregation	Fluorescence (Em460)	Solution	42 × 10^−9^ m	96
C‐dot	CD‐Hg^2+^/Ag^+^	7.6	Quench of the fluorescence	Fluorescence (Em440)	Solution	0.32/0.43 × 10^−6^ m	97
	Ag–C‐dot	≈5	Electrochemiluminescence	Electrochemistry	Solution	0.027 × 10^−6^ m	51
	GBR	N/A	Catalyst	Colorimetry (A652)	Solution	25.3 × 10^−6^ m	98
	PPy/WO_3_	50–70	Reduction	Electrochemistry	Solution	100 ppb	99
	SnO_2_/rGO	≈4	Chemisorption	Electrochemistry	Gas	43 ppb	100
Others	Cyclen‐FPNs	33–40	Metal precipitation	Fluorescence (Em540)	Solution	2.1 × 10^−6^ m	101
	Pb^2+^‐MoS_2_ nanosheet	N/A	Quench of the fluorescence	Fluorescence (Em406)	Solution	0.42 × 10^−6^ m	102
	PbO/SiO_2_	50–100	Quench of the fluorescence	Fluorescence (Em510)	Solution	0.138 × 10^−6^ m	103
	Ru NPs	1.7 ± 0.2	Catalyst	Colorimetry (A512)	Solution	0.6 × 10^−9^ m	44

^a)^ LSPR: localized surface plasmon resonance; NCs: nanocluster; NRs: nanorods; NDs: nanodots; NWs: nanowires; NNs: nanoneedles.

### 3.1 Noble Metal Nanomaterials

Gold and silver are two major noble metals that have been widely used in daily life for centuries. For instance, colloidal gold is a well‐known dye for glass staining that can be traced back to the Roman era. With the excellent stability, catalytic ability, and optical properties, gold, silver, and alloy nanomaterials have been widely developed and applied for biomedical engineering applications.^43^, ^85^, ^87^ The LSPR is a key characteristic of noble metal nanomaterial that is easily influenced by the size, distance, and composition.^104^ Based on this property, a variety of detection methods have been developed by the formation/dispersion of aggregation or change of the surface, including the specific detection for hydrogen sulfide (Table 2).

**Figure 2:**
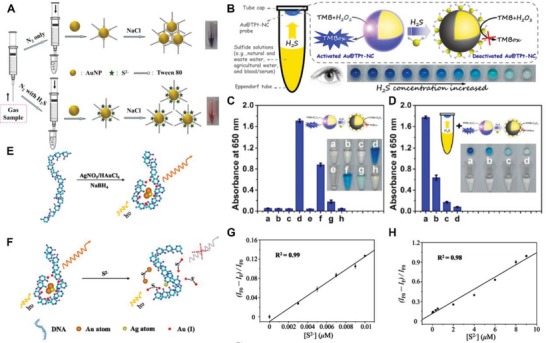
A) The scheme of AuNPs for detecting bubbling H_2_S with the coordination of NaCl and Tween‐80. Reproduced with permission.^86^ Copyright 2014, American Chemical Society. B) Schematic demonstration of the Au@TPt‐NCs‐based platform for detecting dissolved hydrogen sulfide via a colorimetric strategy. C) The deactivated assays of Au@TPt‐NCs (Au core @ ultrathin platinum shell nanoclusters) via H_2_S (the testing groups included: a) Au@TPt‐NCs; b) H_2_O_2_ + TMB (3,3′,5,5′‐tetramethylbenzidine); c) 0.1 × 10^−6^ m H_2_S; d) Au@TPt‐NCs + H_2_O_2_ + TMB; e) Au@TPt‐NCs + 0.1 × 10^−6^ m H_2_S; f) Au@TPt‐NCs + 0.1 × 10^−6^ m H_2_S + H_2_O_2_ + TMB; g) Au@TPt‐NCs + 0.5 × 10^−6^ m H_2_S + H_2_O_2_ + TMB); h) Au@TPt‐NCs + 1 × 10^−6^ m H_2_S + H_2_O_2_ + TMB); D) the detection of H_2_S at various concentrations (a) 0 × 10^−6^ m; b) 0.1 × 10^−6^ m; c) 0.2 × 10^−6^ m; d) 0.5 × 10^−6^ m) via the catalysis of Au@TPt‐NCs platform (*n* = 3). Reproduced with permission.^87^ Copyright 2015, American Chemical Society. E,F) Schematic illustration of DNA‐Au/Ag NCs Probe's synthesis and detection of H_2_S; H_2_S‐induced fluorescent quenching of DNA‐Au/Ag NCs in the presence of S^2−^ ions over G) 0 × 10^−6^ –0.01 × 10^−6^ m and H) 0.01 × 10^−6^–9 × 10^−6^ m. Reproduced with permission.^89^ Copyright 2011, American Chemical Society.

With proper surface functionalization using different ligands such as glutathione,^77^ fluorosurfactants,^82^ or small molecules (e.g., thiolated azido derivates and active esters),^44^ gold nanosensors quickly aggregate when they encounter with H_2_S. This results in a redshift of absorbance wavelengths and LOD ranging from 0.2 × 10^−6^ to 20 × 10^−6^ m. In comparison, hydrophobic surface modification (e.g., fluorescent probe, 1‐(10‐mercaptodecyl)‐5‐methylpyrimidine‐2,4‐dione, TSH) force the AuNDs coated with TSH and MUA (11‐mercaptoundecanoic acid) to aggregate. The presence of H_2_S could disassemble the aggregation surface adsorption of H_2_S and HS, recovering the quantum yield back to 1.61%.^79^ Similarly, Zhang et al. developed a simple sensing strategy by using bubbling H_2_S to stabilize the AuNPs (13 nm), with the existence of NaCl (80 × 10^−3^ m) and Tween 80 (**Figure** **2**A).^84^ This cost‐effective method provides a high sensitivity toward H_2_S with LOD values reaching around 14 × 10^−6^ m for the naked eye and 30 × 10^−9^ m for machine detection, which is more efficient than that afforded by TSH‐MUA‐AuNDs (0.5 × 10^−6^ m).

While other approaches, such as the change of LSPR induced by surface reduction and competitive binding between S‐Au and I‐Au (forming clusters or larger nanoparticles),^80^, ^81^ have been used with gold‐based sensors, the sensing limits only reach about 0.3 × 10^−6^ m for H_2_S detection. Comparably, catalysis mediated by Au based nanosensors has excellent sensitivity.^83^, ^85^ A catalysis Au@TPt‐NCs (Au core with an ultrathin platinum shell) nanoplatform was developed by Gao et al. to detect dissolved H_2_S gas (Figure 2B).^85^ The H_2_S evaporated or dissolved interacts with and deactivates the nanoclusters, attenuating the chromogenic reaction between H_2_O_2_ and 3,3′,5,5′‐tetramethylbenzidine (TMB) and showing an extremely low LOD value at 7.5 × 10^−9^ m. More importantly, the approach is also visible to the naked, providing flexibility for applications (Figure 2C,D).

Additionally, the Au/Ag alloy has also been recruited for sensing H_2_S by fluorescence quenching.^86^, ^87^ Among all, a sensitive DNA‐templated Au/Ag NCs was successfully developed by Chen et al (Figure 2E,F).^87^ In the presence of H_2_S, the prepared Au/Ag NCs showed a linear relationship (0 × 10^−6^–0.01 × 10^−6^ m and 0.01 × 10^−6^–9 × 10^−6^ m) between the H_2_S concentration and fluorescence intensity, with a quantum yield of 4.5% and a LOD of 0.83 × 10^−9^ m (Figure 2G,H). Among Ag‐based nanoplatforms, only several polymer‐coating Ag nanoparticles have been investigated and relatively‐low efficiency was demonstrated for H_2_S detection (0.2 × 10^−6^–3.3 × 10^−6^ m) compared with that provided by Au‐based nanoprobes.^42^, ^43^, ^88^

### 3.2 Metal‐Organic Framework (MOF)

**Figure 3:**
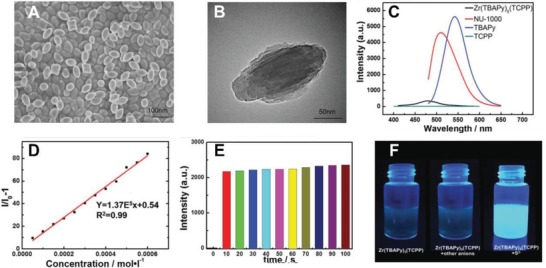
A) Representative TEM and B) HR‐TEM images of Zr(TBAPy)_5_(TCPP); C) The photoluminescence emission spectra among Zr(TBAPy)5(TCPP) (black), NU‐1000 (red), TBAPy (blue), and TCPP (cyan). D) The variation of fluorescence generated by Zr(TBAPy)5(TCPP) with a series of S^2−^ concentrations and E) the change of fluorescence intensity at various time points post the addition of S^2−^ into the Zr(TBAPy)5(TCPP) solution; F) Fluorescence pictures (λ_ex_ = 365 nm) of Zr(TBAPy)5(TCPP) aqueous solutions with different anions. Reproduced with permission.^93^ Copyright 2018, Wiley.

The past decade has seen drawn a great deal of attention to metal‐organic framework (MOF) due to their excellent physiochemical features.^105^, ^106^, ^107^, ^108^, ^109^, ^110^ These nanomaterials are composed of different combinations of metal ions, organic linkers, and modifications and have vast application possibilities (e.g., gas storage, chemical sensing, chiral separations, etc.).^111^ With the tremendous surface area (≈7000 m^2^ g^−1^) and rigid pores that could host various functional molecules, MOF has also been investigated as a potential sensor for chemical and toxic gas detection, such as hydrosulfide.^112^ Through the formation of the metal sulfides (e.g., CuS),^46^, ^89^, ^90^ amine group,^39^ or N—S bond^91^ with S^2−^, several novel MOFs could recover the fluorescence/luminescence that was quenched and trigger a detectable signal for sensing H_2_S with a desirable sensitivity. For instance, the presence of Tb^3+^/Cu^2+^ ions enables the Tb^3+^@Cu1/Cu2 MOF complex to generate multiwavelength luminescence and produce an enhanced ratiometric signal (*I*_544_/*I*_390_) after the interaction with the H_2_S exposed, with a LOD of S^2−^ at about 1.2 × 10^−6^ m.^89^ Similarly, Qian Lab synthesized an Eu^3+^@UiO‐66‐(COOH)_2_ MOF that induced a fluorescent signal via the interaction between Cu^2+^ and S^2−^.^46^ Although such MOF exhibits a uniform nanostructure (80–100 nm) and comparable H_2_S LOD (5.45 × 10^−6^ m), the fluorescence intensity generated could be affected by amino acids containing thiol and nitroxyl groups, which strongly lowers selectivity toward H_2_S.^46^ Comparably, the novel sensors, Zr(TBAPy)_5_(TCPP) and aluminum‐based MOF (Al‐MIL‐53‐NO_2_) demonstrate desirable H_2_S detection and selectivity via reduction, with LOD of ≈92.31 × 10^−9^ m and ≈1 ppb, respectively.^39^, ^91^ The Zr(TBAPy)_5_(TCPP) were synthesized with a uniform nanostructure (with a diameter around 100 nm) after incorporation of Tris(2‐chloroisopropyl)phosphate (TCPP) (**Figure** **3**A,B).^91^ This synthesized nanoparticle was very sensitive to H_2_S (with a LOD around 50 × 10^−9^ m), and only showed fluorescence after the introduction of H_2_S, demonstrating a desirable linear relation between fluorescence and the concentration of H_2_S (Figure 3C,D,F). More importantly, the reaction of Zr(TBAPy)_5_(TCPP) and H_2_S was completed within 10 s, providing an opportunity for real‐time detection (Figure 3E).

As alternatives to single substrate MOFs, probes for the detection of multiple biomolecules are highly desirable for large scale detection in environmental or clinical assay. Recently, a Eu^3+^‐Cu^2+^ based MOF was developed.^90^ With two specific and separate binding areas for ascorbic acid (AA) and H_2_S, it simultaneously detected both biomolecules. Due to the high sensitivities, the as‐prepared MOF can identify H_2_S and AA concentrations as lower as 130 × 10^−9^ and 55 × 10^−9^ m, respectively. Additionally, desirable recovery rate (94.7–104.1%) was attained in assays using human serum. After incorporating various elements and molecules, novel MOF‐based probes for multiple biomolecule detection have significant promise for biomedical applications.

### 3.3 Copper Based Nanomaterials

**Figure 4:**
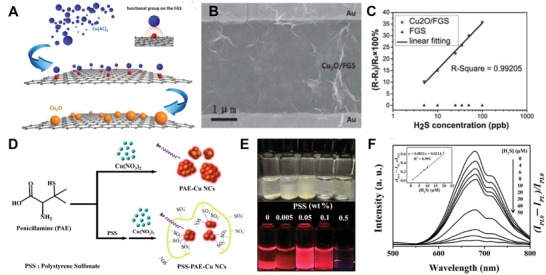
A) Schematic demonstrating the in situ approach for synthesizing the Cu_2_O‐FGS (functional graphene sheets) platform; B) Representative SEM image of the Cu_2_O–FGS established on the Si/SiO_2_ substrate with gold interdigitated electrodes coverage; C) Sensitivity limits of Cu_2_O–FGS and FGS based detector in series of concentrations of atmospheric H_2_S. Reproduced with permission.^52^ Copyright 2013, Royal Society of Chemistry. D) Schematic illustration of the polystyrene sulfonate (PSS) mediated PSS‐PAE‐Cu NC synthesis. E) Optical images of PSS‐PAE‐Cu NC aggregates prepared via various concentrations of PSS (0.005–0.5 wt%) without (upper row) and with UV illumination; F) The photoluminescence spectra of the PSS‐PAE‐Cu NC aggregates under various concentrations of H_2_S and the linear relationship between the photoluminescent intensity of PSS‐PAE‐Cu NC aggregates and the concentration of H_2_S with sodium phosphate buffer (10 × 10^−3^ m, pH 3.0). Reproduced with permission.^97^ Copyright 2016, Nature Research.

Copper (Cu), the most‐used cation for H_2_S sensing (via the metal precipitation), has been widely incorporated into small organic molecules (e.g., HSIP‐1) and the other nanosized probes.^78^, ^80^ The addition of Cu to nanomaterials in the form of Cu, CuO, or Cu_2_O is also employed for H_2_S‐specific detection.^52^ Coating the surface of nanoparticle sensors (e.g., nanowires, nanoneedles, or nanotubes) with Cu, CuO, or Cu_2_O enables rapid detection of H_2_S due to variations in conductivity after reduction. As such, the concentration of H_2_S in the solution or air can be determined.^53^, ^92^, ^93^ For example, a Cu_2_O NPs (2–3 nm) coated WO_3_ nanoneedles were prepared via aerosol‐assisted chemical vapor deposition (AACVD).^53^ This system was able to detect H_2_S levels as low as 300 ppb within two seconds. A major limitation of the Cu_2_O‐WO_3_ nanoneedles was the high temperature required (390 °C) that makes practical application difficult. A Quasi‐2D‐Cu_2_O/SnO_2_ consisting of P‐type Cu_2_O and N‐type SnO_2_ was successfully developed for H_2_S gas detection at room temperature, with a LOD at 0.5 ppm.^94^ Notably, laser illumination further reduced the heterojunction barrier and enhanced the response of Quasi‐2D‐Cu_2_O/SnO_2_ by 20%. The Chen lab synthesized a Cu_2_O‐FGS (functional graphene sheets) by in situ growth that provided desirable surface accessibility, contacting area (Cu_2_O was prepared without surfactant) and sensitivity (LOD is around 5 ppb) for H_2_S gas sensing under normal atmospheric conditions (**Figure** **4**A–C).^52^

Several studies have confirmed that the aggregation of organic Cu NCs (e.g., cysteine or penicillamine (PAE) template) can activate an enhanced fluorescence referred to as aggregation‐induced emission (AIE).^95^, ^96^ By incorporating polystyrene sulfonate (PSS) into the system, PSS‐PAE‐Cu NCs aggregates were designed H_2_S detection in drinking water (Figure 4D).^95^ With the 0.05 wt% PSS, as‐prepared PSS‐PAE‐Cu NCs aggregates (173 nm) generated red photoluminescence (665 nm) that was extinguished when as little as 650 × 10^−9^ m H_2_S was present (Figure 4E,F).

### 3.4 Carbon Nanodot

Since the first discovery at 2004, carbon nanodots (C‐dots or CDs) have been widely investigated for biomedical, catalytic, and sensing applications due to its attractive features of high solubility, biocompatibility, and photostability.^113^, ^114^, ^115^ Among all, several novel C‐dots have been designed for H_2_S detection/imaging.^51^, ^97^, ^116^, ^117^ For example, two metal ion (Ag^+^/Hg^+^) based C‐dots were synthesized for sensing sulfide ions. In the presence of H_2_S as low as 0.32 × 10^−6^ and 0.43 × 10^−6^ m respectively, the fluorescence of CD‐Hg^+^/Ag^+^ would be quenched by the inner filter effect (IFE) mediated by the Hg_2_S/Ag_2_S formed. Meanwhile, the formation of Ag_2_S significantly changes the Ag–C‐dot's electrochemiluminescence that shows a desirable sensitivity with a LOD at 27 × 10^−9^ m.^56^, ^57^

### 3.5 Other Nanosensors

**Figure 5:**
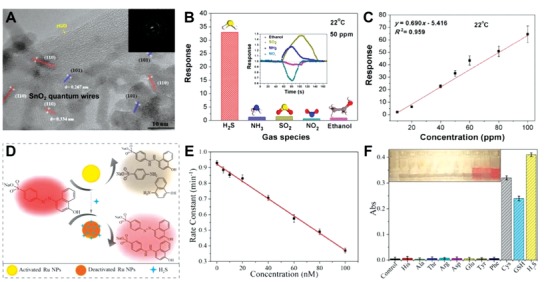
A) Representative HR‐TEM images of SnO_2_/rGO nanocomposites and the electron diffraction pattern within the selected area. B) The selective capability of SnO_2_/rGO nanocomposites for detecting H_2_S gas among various interference. C) The response rate of SnO_2_/rGO nanocomposites under different H_2_S gas concentrations. Reproduced with permission.^102^ Copyright 2016, American Chemical Society. D) Schematic demonstrating the Ru NPs‐based colorimetric assay for H_2_S detection via the hydrogenation catalysis. E) The change of constant rate under various concentrations of Na_2_S (5 × 10^−9^–100 × 10^−9^ m), the H_2_S donor. F) The different absorbance intensities (512 nm) of Ru‐NPs and orange I mixtures with various biological thiols and other amino acids attendance, and corresponding optical images of samples within 2 min (inserted figure). Reproduced with permission.^44^ Copyright 2017, American Chemical Society.

Other nanomaterials such as Pb‐based NPs, graphene supporting and polymeric nanocomposites, and ruthenium NPs have been investigated as nanosensors for H_2_S detection (Table 2).^44^, ^98^, ^99^, ^100^, ^101^, ^102^, ^103^ Given the great conductivity of graphene, a SnO_2_‐rGO (reduced graphene oxide) nanosheet was successfully developed via a one‐step colloidal synthesis for H_2_S sensing (**Figure** **5**A).^100^ H_2_S gas was adsorbed (i.e., chemisorption) and detected within 2 s at room temperature with a desirable sensitivity (with LOD at 43 ppb) (Figure 5B,C). Two polymeric nanoparticles, the PPy/WO_3_ (50–70 nm) and cyclen‐FPNs (33–40 nm) were designed for identifying this gas as well.^99^, ^101^ After electrochemical electron transfer (i.e., H_2_S + 3O_2_^−^ → 2H_2_O + 2SO_2_ + 3e^−^) and the formation of CuS (i.e., the recovery of fluorescence), H_2_S concentrations could be well determined by PPy/WO3 and cyclen‐FPNs (Table 2). Recently, Zhao et al. developed a colorimetric approach via the catalysts mediated by ruthenium nanoparticles (Ru NPs) (Figure 5D).^44^ The synthesized Ru NPs (1.7 ± 0.2 nm) degraded the organic dye—Orange I. The resulting color fade occured about 4, 47, and 165 times faster than for platinum (Pt), iridium (Ir) based NPs, and control groups. Exposure of the Ru NPs and Orange I to H_2_S protected the Orange I by deactivating Ru NPs (Figure 5E). The superior catalytic capability of Ru NPs demonstrated an excellent LOD (about 0.6 × 10^−9^ m), but it had a relatively poor selectivity to H_2_S as cross‐reaction with Cys and GSH occurred (Figure 5F).^44^

## 4 H_2_S Imaging with SHTS‐Based Nanomedicine

As we have mentioned, micro‐ or nano‐probes have been widely applied for H_2_S measurements in clinical samples and has greatly facilitated bench efforts. However, the real‐time imaging of H_2_S secretion in patients for disease diagnosis, especially tumor tracking, is still highly demanded. Among all the in vitro and in vivo imaging candidates, nanocarriers have shown great potential as fluorescent, LSPR, upconversion luminescence (UCL), near infrared (NIR), photoacoustic (PA) and positron emission tomography (PET) imaging probes (**Table** **3**). In comparison with the fluorescence and UCL imaging, only a few of nanoprobes has been used for H_2_S imaging via NIR, LSRP, PA, and PET. These nanosensors would be described in detail in this section.

**Table 3:** The novel nanosensors as SHTS for H_2_S imaging

Imaging strategy^a)^	Material	Nanoparticles	Size [nm]	Mechanism	Assay	Sample(s)	LOD	Ref.
FL	MOF	UiO‐66‐CH = CH2	20–30	Reduction (C = C)	Em ≈ 370	PC‐12 cell	6.46 × 10^−6^ m	120
		[Al(OH)(IPA‐N3)]·3.2 H_2_O·0.4DMF	N/A	Reduction	Em405	J774A.1 cell	2.65 × 10^−6^ m	121
		CuO@TO@UiO‐66	N/A	FRET interruption (Turn‐off)	Em520‐650	A549 and HepG2 cell	0.51 × 10^−6^ m	45
	C‐dot	C‐Dot‐Ligand‐Cu^2+^	≈5	Metal precipitation	Em455	HeLa and L929 cell	0.78 × 10^−6^ m	117
		C‐Dot‐TPEA–Cu^2+^	≈5	Metal precipitation	Em560	Hela cell and A549 tumor slide	0.7 × 10^−6^ m	116
		Cyclam‐CDs (CCDs)	≈2	Metal precipitation	Em460	Hela cell	130 × 10^−9^ m	122
		FCDs‐Cu^2+^	4	Metal precipitation	Em452	Hela cell	88.9 × 10^−9^ m	40
		CD‐based sensor	≈5	FRET induction (Turn‐on)	I526/I425	HeLa and L929 cell	10 × 10^−9^ m	50
	Other	FAM‐DNA/AgNP	10 ± 3	Reduction	Em520	Hela cell	10 × 10^−9^ m	47
		NanoBODIPY	≈10	FRET interruption (Turn‐on)	Em589	Raw 264.7 cell	7 × 10^−9^ m	49
LSPR	Au/Ag	Au/Ag PNPs	74.19	LSPR shift	A702 (Dark field imaging)	HepG2 and Hela cell	0.1 × 10^−6^ m	41
UCL	UCNPs	Cy7‐UCNPs	11.27–44.6	LRET interruption (Turn‐on)	Em800	Hela and MCF‐7 cell; Zebra fish	510 × 10^−9^ m	123
		TPAMC‐UCNPs@PEG	≈35	LRET interruption (Turn‐on)	I530/I660	Hela and MCF‐7; HCT‐116 bearing mice	0.22 × 10^−6^ m	48
		NaYF4: 20% Yb, 2% Er, 0.2% Tm	94	LRET interruption (Turn‐on)	I_green_/I_red_	Hela cell	0.58 × 10^−6^ m	124
		CHC_1_‐UCNPs	24	LRET interruption (Turn‐on)	*I*_541_/*I*_800_	Hela cell and Mice (LPS)	0.13 × 10^−6^ m	125
		PAA‐NaYF 4:Yb/Er/Tm	12	LRET interruption (Turn‐on)	I540/I800 or I650/I800	Hela cell	N/A	34
NIR	Silica	ZX‐NIR	≈66	Nucleophilic substitution	Em900‐1300	HepG2, HCT‐116 cell and tumor‐bearing mice	≈37 × 10^−9^ m	126
PA	Silica	Si@BODPA180	≈75	Nucleophilic substitution	ex780	HCT‐116 bearing mice	53 × 10^−9^ m	38
	Liposome	AzHD‐LP	≈12	Reduction	ex700	HCT‐116 cells and HCT‐116 bearing mice	91 × 10^−9^ m	127
PET	^64^Cu^2+^	^64^Cu‐cyclen	N/A	Metal precipitation	PET	Mice and Rat	0.15 × 10^−6^ m (≈1% g^−1^)	128

^a)^ FL: fluorescence; UCL: Upconversion luminescence; NIR: Near infrared; PA: photoacoustic imaging; PET: positron emission tomography.

### 4.1 Fluorescence Imaging

With the innovation of imaging technology, two‐ and multiphoton microscopy has been used for fluorescence imaging, which greatly improved the depth of penetration (≈1 mm).^118^, ^119^ However, most fluorescent agents have generally employed for living cell or tissue section based imaging. For instance, the incorporation of organic components (azide or unsaturated C=C bond) enabled MOF to be used for specific imaging in a series of cancer cell lines (e.g., PC12 and J774A.1 cells) via reduction mediated by H_2_S.^120^, ^121^ Comparably, further functionalization with Cu^2+^ based ligands (e.g., Cyclam‐Cu^2+^) enabled the C‐Dots to visualize H_2_S within cells via fluorescence initiated after CuS precipitation.^40^, ^116^, ^117^, ^122^ These nanosensors demonstrated desirable biocompatibility and efficiently detected H_2_S in Hela or L929 cells, with LOD ranging from around 90 × 10^−9^–780 × 10^−9^ m. Notably, C‐Dot‐TPEA–Cu^2+^, a two‐photon nanoprobe, exhibited excellent tumor penetration that could be used for sensing H_2_S in A549 tumor sections. This system provided an emission wavelength (560 nm) suitable to minimize background for H_2_S and nuclei imaging, compared with those (≈460 nm) offered by other C‐dots.^116^

**Figure 6:**
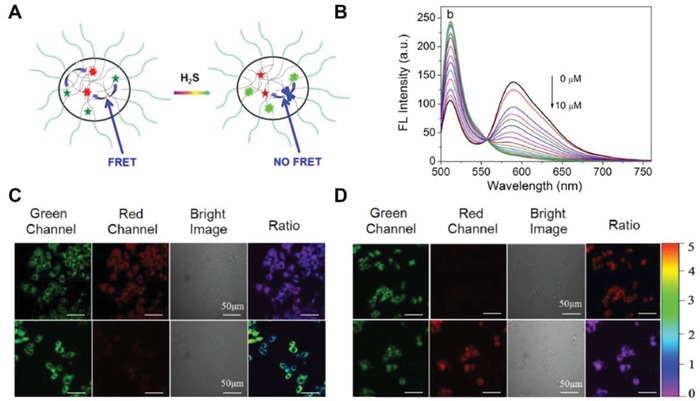
A) Schematic interpretation of FRET between the responsive energy acceptor (BODInD‐Cl, red star) and the complementary energy donor (BODIPY1, green star) in the NanoBODIPY micellar aggregate. B) The fluorescence spectra of the NanoBODIPY under different concentrations of NaHS (0 × 10^−6^ to 10.0 × 10^−6^ m); confocal microscopy images detecting H_2_S within live macrophage cells (Raw 264.7) using NanoBODIPY. C) 30 min incubation of cells and NanoBODIPY (top row) and 30 min incubation of the cells pretreated with 2.0 × 10^−6^ m fluvastatin (the CSE stimulator) for 48 h and NanoBODIPY (bottom row). D) 30 min incubation cells and NanoBODIPY followed by 100 × 10^−6^ m NaHS (top row) and 30 min incubation of the cells pretreated with 1 × 10^−3^ m DL‐propargylglycine (PAG, the CSE inhibitor) for 1 h along with 2.0 × 10^−6^ m fluvastatin (the CSE stimulator) for 48 h and NanoBODIPY (bottom row); ratio images were generated for the green channel (500–550 nm) relative to the red channel (560–650 nm). Reproduced with permission.^49^ Copyright 2016, American Chemical Society.

Internal Förster resonance energy transfer (FRET) is able to aid specific imaging of H_2_S in vivo. The FRET acceptor (e.g., CuO coated on the surface) or probe structure changes could initiate or terminate FRET in response to H_2_S.^45^, ^49^, ^50^ Notably, carried fluorophores can change its excitation or emission wavelength to act as the imaging trigger when exposed to H_2_S.^49^ For instance, boron‐dipyrromethene (BODIPY), with a small Stokes shift and high fluorescent quantum yields, has been widely employed in various nanosized platforms for probing H_2_S. A micellar nanomaterial was designed by incorporating an amphiphilic copolymer (mPEGDSPE), semi‐cyanine‐BODIPY hybrid dye (BODInDCI), and BODIPY1 as the energy donor for H_2_S imaging. (**Figure** **6**A).^49^ Once the BODInDCl was exposed to H_2_S, its absorption wavelength rapidly shifted from 540 to 738 nm and suspend FRET between it and BODIPY1, eventually recovering and switching off fluorescence at 511 and 589 nm (Figure 6B). Importantly, this reaction was quickly finished within 140 s, demonstrating high efficiency for H_2_S detection.^129^ Additionally, this nanoBODIPY probe was able to track endogenous H_2_S in a macrophage cell line (RAW 264.7) based on the ratio between the dual‐color images (Figure 6C,D).^49^

### 4.2 LSPR Dark‐Field Imaging

**Figure 7:**
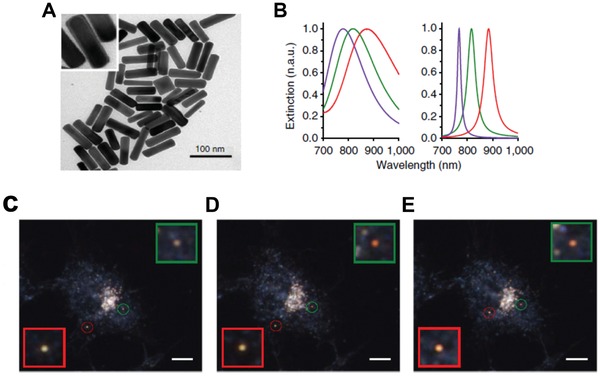
A) TEM and HR‐TEM images of AuNR‐Ag nanoprobes consisting of a AuNR core and Ag shell. B) The LSPR spectra of AuNR (green), AuNR‐Ag (purple), and AuNR‐Ag_2_S (red) nanoparticles via the experimental (left) and discrete dipole approximation (DDA) simulated approaches (right). The representative dark‐field images of two individual PNPs in different time points at C) 2 min, D) 26 min, and E) 42 min after the addition of 0.1 × 10^−3^ m Na_2_S into the HepG2 cells. The length of the scale bar is 10 mm. The individual PNPs are enlarged and presented in the inserted squares. Reproduced with permission.^41^ Copyright 2013, Nature Research.

Plasmonic nanoparticles (PNPs), such as gold nanorods (AuNR), can provide extremely bright signal compared with organic fluorescent dyes.^130^, ^131^ By further coating Ag on the AuNR, Xiong et al. successfully applied the gold nanorod‐silver (AuNR‐Ag) core–shell PNPs for mapping H_2_S *n* living cells via dark‐field imaging.^41^ The AuNR‐Ag PNP (74 × 19 nm core and 2.1 nm shell) generated Ag_2_S and changed its LSPR wavelength when it encountered with H_2_S (**Figure** **7**A,B). Notably, a linear logarithmic was observed between the spectral shifts and sulfide concentrations (ranging from 0.01 nm to 10 × 10^−6^ m) at various time points (1–30 min), indicating extremely high sensitivity. In addition, the AuNR‐Ag PNP demonstrated excellent H_2_S selectivity compared to other inorganic sulfur ions. Using this nanoplatform, the fluctuations of sulfide (0 × 10^−9^–100 × 10^−9^ m) and real‐time H_2_S mapping/calculation around single AuNR‐Ag PNP within live cells (from 5.8 × 10^−9^–41.8 × 10^−9^ m or 0.5 × 10^−9^–3.8 × 10^−9^ m for P1 or P2 respectively) was successfully achieved (Figure 7C–E).

### 4.3 UCL Imaging

Upconversion nanoparticles (UCNPs) convert continuous‐wave (CW) NIR wavelengths to visible light with a sizeable anti‐Stokes shift of several hundred nanometers.^132^, ^133^, ^134^, ^135^, ^136^, ^137^ Compared to organic dyes and inorganic semiconductor nanoparticles, UCNPs display superior features, such as scarcely autofluorescence from biological samples,^138^ a remarkable light penetration depth (up to 10 mm),^139^ no photobleaching in bioapplications,^140^, ^141^ and less damage to biological samples than UV excitation source.^142^ As a result, UCNPs are ideal probes for visualizing living cells and whole‐body animals.^143^, ^144^, ^145^ To achieve a sensing function, UCNPs need to combine with other chromophores with recognition sites, through the luminescence resonance energy transfer (LRET) process. Several UCNPs‐chromophores based LRET nanosystems have been developed for detecting critical biological species and toxins, such as DNA, O_2_, CN^−^, Hg^2+^, and Zn^2+^.^146^, ^147^, ^148^, ^149^, ^150^ In these applications, the UCNPs (donor) transfer energy to the organic chromophores (acceptor) and results in changes to the UCL emission. Thus, UCNPs^‐^chromophores are excellent candidates for H_2_S sensing probes.

**Figure 8:**
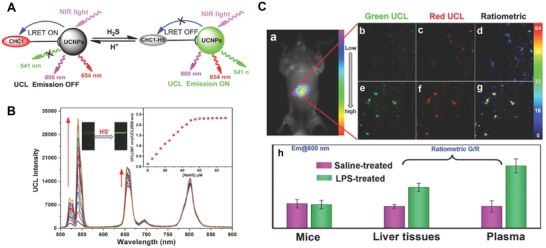
A) Schematic demonstrating the LRET process between the energy acceptor (CHC1) and energy donor (UCNPs). B) The change of CHC1‐UCNPs' UCL emission patterns under a series of H_2_S concentrations (0 × 10^−6^–90 × 10^−6^ m), and the ratiometric values (*I*_541_/*I*_800_) along with optical image of green UCL emission are presented as inserted figure and photo. C) The UCL imaging of H_2_S expressed endogenously in mouse liver: a) the in vivo UCL imaging of the mice with inflammatory (for 24 h) after intravenous injection with CHC1‐UCNPs; b–d) The UCL images of liver section obtained from the mice injected with PBS and CHC1‐UCNPs only; e–g) The UCL images of liver section harvested from the mice administrated with LPS (20 mg kg^−1^) and CHC1‐UCNPs; h) The average UCL ratiometric values among different tissues. Images were acquired under an excitation 980 nm, with a green channel around 500–560 nm and red channel at 600–700 nm. Reproduced with permission.^153^ Copyright 2014, Wiley.

Since the multicolor luminescence of UCNPs can be tuned by doping different ions, a series of chromophores with different absorption bands could be combined and designed for H_2_S‐specific response. For example, Peng et al. compared three H_2_S‐responsive chromophores combined with different doping ions UCNPs (NaYF_4_:Yb/Er/Tm, NaYF_4_:Yb/Er/Mn). This library of H_2_S sensors had responsive emission signals ranging from the visible to the NIR region.^34^ These UCNPs‐chromophores showed various LRET efficiency (11.8–25.1%), but all exhibited high excellent selectivity and rapid responsiveness in live cells and blood serum. Doping Tm^3+^ into UCNPs introduces UCL signals at 800 nm that can be utilized as an internal standard for ratiometric detection of H_2_S to improve sensitivity. As an example, Liu et al. employed NaYF_4_:20%Yb,2%Er,0.2%Tm@mSiO_2_‐merocyanines for ratiometric detection of H_2_S using multiwavelength UCL.^151^ UCNPs@mSiO_2_‐MC showed an enhanced ratiometric signal (*I*_540_/*I*_800_) for higher sensitivity with LOD at ≈0.58 × 10^−6^ m, which was lower than that of another merocyanine‐based H_2_S probe (1.0 × 10^−6^ m).^152^ Similarly, Zhou et al. used NaYF_4_:20%Yb,1.8%Er,0.5%Tm@α‐cyclodextrin (CD)–coumarin hemicyanine (CHC1) dye as a ratiometric UCL probe (**Figure** **8**A).^153^ By measuring the ratio of *I*_580_/*I*_800,_ this UCNPs was able to measure H_2_S concentrations as low as 0.13 µm, much more sensitive than single UCL signals (1.85 µm) in aqueous solution (Figure 8B). This UCNPs@CD‐CHC1 could be used for ratiometric UCL monitoring of pseudo‐enzymatic H_2_S production in living cells, and also showed for ability to detect lipopolysaccharide (LPS)‐induced inflammation in the liver tissues of mouse models for the first time (Figure 8C).

**Figure 9:**
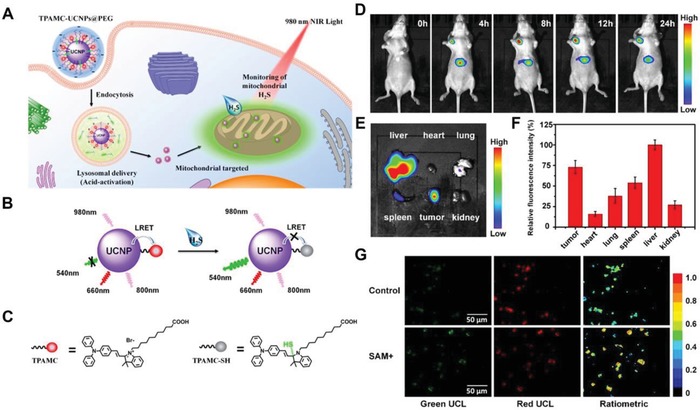
A) Schematic of the cellular targeting process and mitochondrial H_2_S detection mediated by the TPAMC‐UCNPs@PEG nanoplatform. B) Schematic of the LRET mechanism between UCNPs and TPAMC with or without H_2_S. C) The chemical structures of TPAMC and TPAMC‐SH. D) The UCL imaging obtained from HCT116 tumor‐bearing mice post‐intravenous administration of TPAMC‐UCNPs@PEG. E) The ex vivo UCL images and F) the corresponding fluorescence quantifications of the major organs at 24 h post the TPAMC‐UCNPs@PEG injection (*n* = 3). G) The two single channels and ratiometric UCL images of the tumor sections from the mice intravenously administrated with TPAMC‐UCNPs@PEG and further injected with PBS (top raw) and S‐adenosyl‐l‐methionine (SAM) (button raw). The green (500–560 nm) and red (600–680 nm) signal of UCL were obtained under a 980 nm excitation. Reproduced with permission.^156^ Copyright 2018, American Chemical Society.

UCNPs have also been developed for detecting or imaging of small molecules, biomacromolecules, organs, and tumors. Li et al. developed a merocyanine derivative modified UCNPs (NaYF_4_: 20%Yb, 2%Er, 0.2%Tm)@PEG as a ratiometric UCL probe for H_2_S detection in mitochondria of live cells and live‐tissues (**Figure** **9**A–C).^154^ This probe was used for locating the HCT116 (human colorectal cancer cell line) tumor in vivo by using NIR UCL imaging (Figure 9D–F). Additionally, this system was capable of monitoring mitochondrial H_2_S within tumor slices via a ratiometric UCL measurement (Figure 9G).

**Figure 10:**
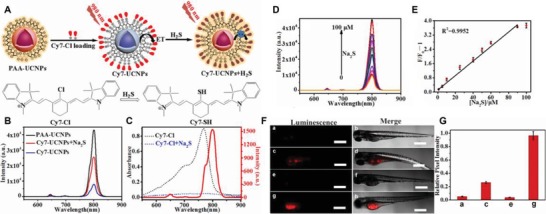
A) Schematic of luminescent strategy employed by PAA‐UCNPs, and the chemical structures of Cy7‐Cl and Cy7‐SH. B) The luminescence spectra of PAA‐UCNPs, Cy7‐UCNPs, and Cy7‐UCNPs + Na_2_S (50  × 10^−6^ m). C) The UV–vis absorption spectra of Cy7‐Cl (black) along with Cy7‐Cl + Na_2_S (blue), and the UCNPs' luminescence spectrum (red). D) The change of luminescence spectra upon the addition of various Na_2_S concentrations (0 × 10^−6^–100  × 10^−6^ m). E) The enhancement of fluorescence ratio accompanied by increasing concentrations of Na_2_S. F) In vivo UCL images of exogenous and endogenous H_2_S in zebrafish via the Cy7‐UCNPs imaging system: a,b) normal zebrafish were injected with PBS, followed by an administration of Cy7‐UCNPs 30 min later; c,d) tumor‐bearing zebrafish was administrated with PBS, followed by an injection of Cy7‐UCNPs 30 min later; e,f) tumor‐bearing zebrafish was first injected with NMM (the scavenger of intracellular H_2_S), followed by an injection of Cy7‐UCNPs 30 min later; g,h) tumor‐bearing zebrafish was administrated with l‐Cys (the precursor of H_2_S), followed by the administration of Cy7‐UCNPs 30 min later; the length of scale bar is 500 µm. G) The corresponding average UCL intensities of data in (a,c,e,g). Reproduced with permission.^157^ Copyright 2018, Elsevier.

To monitor H_2_S using UCL imaging both ex vivo and in vivo, Wang et al. proposed a PAA‐UCNPs (NaYF_4_:Yb/Tm@NaYF_4_) loaded with a cyanine chromophore (Cy7‐Cl) as a NIR probe for H_2_S response. (**Figure** **10**A).^155^ This nanoprobe was able to emit luminescence at 800 nm (Figure 10B,C) and demonstrated superb sensitivity toward H_2_S (Figure 10D,E). In addition to imaging exogenous and endogenous H_2_S in living cells (Hela and MCF‐7 cells), the Cy7‐UCNPs were successfully employed for sensing H_2_S in tumor‐bearing zebrafish in real time, with high penetration depth and low autofluorescence background (Figure 10F,G). Thus, the UCNPs‐chromophores were capable of monitoring H_2_S in living cells and small animals by UCL imaging. Ratiometric UCL‐based nanosystems provide a new design strategy for sensing and imaging of H_2_S that might be further utilized by novel probes for highly sensitive in vivo imaging studies.

### 4.4 NIR Imaging

Various fluorescent probes have been successfully employed for detection of cellular H_2_S. However, most of these fluorescent probes emit in the ultraviolet or visible light region (450–750 nm that is impeded by cell autofluorescence. In contrast, long wavelength probes with emission in the NIR region are optimal for biological imaging applications due to minimal photodamage to biological samples and interference from background autofluorescence in living systems.^156^, ^157^ Additionally, NIR light (700–900 nm) can well improve the tissue depth penetration for in vivo imaging.^158^, ^159^ Among NIR fluorochromes, cyanine dyes have excellent photophysical properties, such as outstanding biocompatibility and low toxicity to living systems, which is suitable incorporation as a fluorescent probe.^159^, ^160^ For example, Wang et al. designed a NIR fluorescent cyanine probe Cy–NO_2_ (em. ≈789 nm) for H_2_S detection (via nitro group reduction) in aqueous solution and living cells.^161^ Similarly, Zhang et al. reported a cyanine‐based NIR probe (em. ≈796 nm) for a highly sensitive (with LOD at 39.6 × 10^−9^ m) and selective imaging of endogenous H_2_S in tissues and tumor models (HCT116 and HT29) of mice.^162^

**Figure 11:**
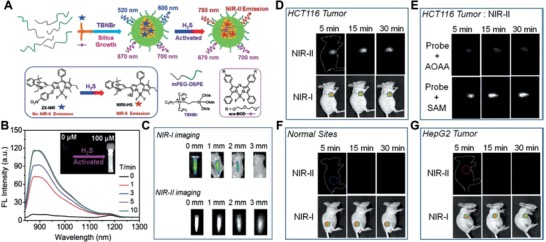
A) Schematic of the formation of NIR‐II@Si nanoprobe and the chemical structures of components including ZX‐NIR and NIRII‐HS. B) The variation of NIR‐II spectra of as‐prepared NIR‐II@Si at different time points after the addition of 100 × 10^−3^ m NaHS, and the NIR‐II images of NIR‐II@Si after H_2_S activation (10 mm ZXNIR) in the presence of 100 mm NaHS (inserted photo). C) The NIR‐I and NIR‐II fluorescent images of the H_2_S‐activated NIR‐II@Si covered by pork skin with various thicknesses. The NIR‐I and NIR‐II imaging of the D) HCT116 tumor, G) HepG2 tumor, and F) normal tissue from tumor‐bearing mice or normal mice at different time points (5, 15, and 30 min) after intratumor or on‐site injection of NIR‐II@Si nanoprobe. E) The NIR‐II images of HCT‐116 tumor‐bearing mice at 5, 15, and 30 min postinjection of NIR‐II@Si nanoprobe along with AOAA (amino‐oxyacetic acid, the inhibitor) or SAM (S‐adenosyl‐l‐methionine, the activator). Reproduced with permission.^166^ Copyright 2018, Wiley.

Recent progress has demonstrated that fluorescence imaging in the second near‐infrared window (NIR‐II, 1000–1700 nm) can further improve image contrast at increased tissue depths. Moreover, NIR‐II fluorescence imaging remarkably reduces interference from photon absorption and displays higher in vivo spatial resolution than NIR‐I imaging.^163^ Zhao's group fabricated an H_2_S‐triggered NIR‐II nanoprobe for visualizing colorectal cancers (**Figure** **11**A).^164^ The nanoprobes were comprised of a silica shield and two organic chromophores, a boron‐dipyrromethene dye generating the NIR‐II emission (em. 900–1300 nm) with the presence of H_2_S and an inert aza‐BODIPY dye (em. 700 nm) as the internal reference (Figure 11B,C). The NIR‐II@Si showed a selective identification of H_2_S rich colon cancer cells via a dual color imaging modality (Figure 11D,F,G). Moreover, NIR‐II@Si was further explored for the H_2_S‐triggered NIR‐II imaging with the supporting of SAM (S‐adenosyl‐l‐methionine, the CBS activator) (HCT‐116 tumors), showing enhanced deep tissue penetration and spatial resolution (Figure 11E).^164^

### 4.5 PA Imaging

**Figure 12:**
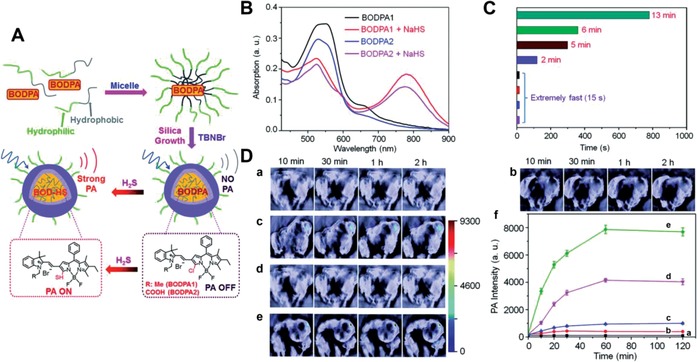
A) Schematic of the Si@BODPA nanoprobe. B) The change of absorbance of the Si@BODPA1 along with Si@BODPA2 with and without NaHS (100 × 10^−3^ m). C) The reaction times among BODPA2‐Si@BODPA30 (13 min), BODPA2‐Si@BODPA90 (6 min), BODPA1‐Si@BODPA30 (5 min), BODPA1‐Si@BODPA90 (2 min), BODPA1/2‐Si@BODPA180/270 (within 15 s), and H_2_S (100 × 10^−3^ m). D) In vivo photoacoustic images of the mice bearing HCT‐116 tumor via the subcutaneously‐injected BODPA1‐Si@BODPA180: a) the tumor regions with saline injection; b) the normal area with nanoprobe injection; c) the tumor regions with nanoprobe administration; d) the tumor area from the pretreated mice (100 nmol AOAA, 12 h in advanced) with nanoprobe administration; e) the tumor area from the pretreated mice (300 nmol SAM, 12 h in advanced) with nanoprobe injection; f) the corresponding PA intensities in a series of time points post BODPA1‐Si@BODPA180 injection. Reproduced with permission.^173^ Copyright 2017, Royal Society of Chemistry.

Among imaging methods that are not fluorescence‐based, PA imaging is a newly emerging technique. This modality is based on the PA effect of translation of excitation light into ultrasonic waves, which bridges the traditional depth and resolution limits of conventional optical imaging techniques.^165^, ^166^ As the acoustic waves are generated by pulsed laser light, noninvasive biomedical images with sharp optical absorption contrast and high ultrasonic resolution are produced.^167^, ^168^ The development of chemical PA probes proposed a new perspective for monitoring therapeutic response and real‐time molecular imaging.^169^, ^170^ For H_2_S detection, Shi et al. first presented a PA probe by encapsulating semi‐cyanine‐BODIPY hybrid dyes into the core–shell silica nanocomposites (Si@BODPA), enabling real‐time imaging of H_2_S‐related biological processes (**Figure** **12**A).^171^ Based on the thiol‐halogen nucleophilic substitution reaction, the Si@BODPA produced emission at 780 nm after the hydrogen sulfide activation, leading to a 44‐fold turn‐on response within 15 s (Figure 12B,C). The LOD was determined to be as low as 53 × 10^−9^ m, a sufficient sensitivity for detecting endogenous H_2_S within living systems. Due to its rapid response, Si@BODPA was then employed for the real‐time monitoring of endogenous H_2_S generation in HCT116 tumor‐bearing mouse to verify elevated level of H_2_S due to CBS upregulation (Figure 12D).

**Figure 13:**
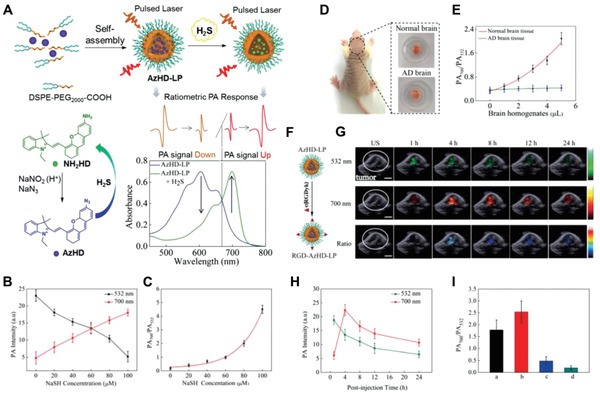
A) Schematic of the ratiometric photoacoustic AzHD‐LP system, the change of the AzHD chemical structures, and PA absorbance (decrease of 532 nm and enhancement of 700 nm) after H_2_S exposure. B) The variation of two PA intensity (532 and 700 nm) of AzHD‐LP under different NaSH concentrations. C) The enhancement of PA700/PA532 ratio with increasing concentration of NaSH. D) The optical images of brain tissue from the normal mice and the mice with Alzheimer's Disease (AD). E) The plot of the PA700/PA532 ratio obtained from the AzHD‐LP after incubation with the brain homogenates from normal or AD mice. F) Schematic of the formation of RGD‐AzHD‐LP. G) The overlayed imaging of PA (PA700 and PA532) or ratiometric PA (PA700/PA532) with ultrasound acquired from the mice bearing subcutaneous HCT116 tumor. H) The corresponding quantitative intensity plot of PA532 (green) and PA700 (red) in (G). I) The ratiometric intensity of PA700/PA532 obtained from different groups at four hours post the intravenous administration of RGD‐AzHD‐LP: a) 12‐hour preinjection of PBS in the tumor area; b) 12‐hour preinjection of SAM (300 nmol) in the tumor area; c) 12‐hour preinjection of AOAA (100 nmol) in the tumor area; d) 12‐hour preinjection of ZnCl_2_ (H_2_S trapper) in the tumor area; the length of scale bar is 5 mm. Reproduced with permission.^129^ Copyright 2018, Royal Society of Chemistry.

Ratiometric PA probes are able to further eliminate some of the shortcomings of a single responsive PA signal by self‐calibration. Thus, the combination of two PA responsive signals at two separated wavelengths would efficiently improve the accuracy of results. For example, Ma et al. developed a ratiometric photoacoustic nanoprobe AzHD (H_2_S‐responsive NIR dye) that was carried by a liposome for monitoring and imaging of H_2_S in cells, brain tissues, and live mice.^127^ With H_2_S‐mediated reduction of the azide, the AzHD‐LP absorption centered at 600 nm gradually decreased, and a new absorption band at 700 nm subsequently appeared (**Figure** **13**A). Through this design, the ratio of PA700/PA532 increased about 4.5‐fold after reactive with H_2_S, which was about 23‐fold higher than a single PA signal alone (Figure 13B,C). The LOD of ratiometric PA signals was determined to be 91 × 10^−9^ m. This enabled the ratio of PA700/PA532 PA signal of healthy and Alzheimer's disease (AD) mice brains (homogenate supernatant) to increase by 6.5 and 1.2‐fold, respectively, following AzHD‐LP introduction (Figure 13D,E). Additionally, further conjugating the RGD targeting group to the AzHD‐LP allowed for successful monitoring of H_2_S in the HCT116 tumor‐bearing mice using time‐dependent dual‐channel ratiometric PA signals (Figure 13F–I). Therefore, the newly designed ratiometric PA probes of H_2_S sensing system provides a powerful analytical and imaging tool for further exploration of the roles of H_2_S in living complex organisms.

### 4.6 PET Imaging

**Figure 14:**
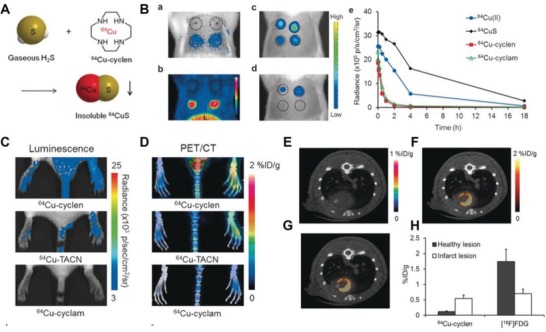
A) Schematic of the formation of ^64^CuS via ^64^Cu–cyclen and H_2_S. B) The detection of H_2_S in vivo: a) Cerenkov luminescence images of the SD rats injected with Matrigel (top left), Matrigel + NaCl (top right), H_2_S gas dissolved in solution (bottom left) and NaHS (bottom right) respectively on the back at 0‐hour postinjection of ^64^Cu‐cyclen; b) The PET image of the injection site at 4 h postinjection (“L” stands for liver); c) The cerenkov luminescence images of SD rats injected with ^64^CuCl_2_ (top left), ^64^CuS (top right), ^64^Cu–cyclen (bottom left), and ^64^Cu–cyclam (bottom right) on the back at 0 h postinjection, and d) 4 h postinjection; e) The clearance of remained sample in a time pattern. C) The cerenkov luminescence images of BALB/c mice with paw inflammation (developed by complete Freund's adjuvant) at 1 h postinjection of various of probes; D) The PET (maximum intensity projection)‐CT images obtained from the BALB/c mice with paw inflammation at 1 h post‐administration of different probes. E) The transverse PET/CT images of the rats with acute myocardial infarction (MI) at 4 h postinjection of ^64^Cu—cyclen. F) The transverse PET/CT images of the rats with MI at 4 h postinjection of ^18^F‐FDG. G) The fused coregisterion image of ^64^Cu–cyclen and ^18^F‐FDG. H) The quantitative analysis of PET imaging of the MI models (*n* = 4). Reproduced with permission.^130^ Copyright 2016, Wiley.

Although fluorescence‐based imaging techniques are primarily utilized for H_2_S detection, their applications in live‐animal imaging are limited because of the limited quantitative analysis. PET provides a highly sensitive non‐invasive technology for molecular imaging assays of metabolism, signal transduction, and gene expression from mice to patients.^172^, ^173^, ^174^ Unsurprisingly, targeted and sensitive PET probes have also been developed for H_2_S imaging. As an example, Yoo's group utilized ^64^CuS nanoparticles for the detection, quantification, and in vivo imaging of endogenous H_2_S via PET imaging.^128^ These nanoparticles were formed by twenty macrocyclic ^64^Cu complexes reacting with gaseous H_2_S to form insoluble ^64^CuS (**Figure** **14**A). ^64^Cu‐cyclen showed high sensitivity (with a LOD at 0.15 µm) and selectivity for H_2_S over other potential competitors, including polysulfides. Due to the physical differences, the intravenously injected ^64^Cu‐cyclen and ^64^Cu‐cyclam were quickly cleared from the body, while the insoluble ^64^CuS nanoparticles were immobilized for more than 4 h after encountering H_2_S (Figure 14B). When ^64^Cu‐cyclen was administrated into mice intravenously, an elevated H_2_S concentration within the inflamed paw was visualized and quantified by both PET imaging and Cerenkov luminescence (Figure 14C,D). Moreover, the ^64^Cu‐cyclen could be also used to detect the defect site in the myocardium from an acute myocardial infarction (MI) model (Figure 14E–H). As such, this radioactive probe demonstrated great potential as a powerful nanoplatform providing efficient detection, accurate quantification, and nuclear imaging of H_2_S within living animals.

## 5 SHTS‐Based Nanomedicine for Disease Therapy

Following disease diagnosis, an effective, timely, and in situ treatment is highly demanded. In comparison to imaging agents, smart nanoplatforms could combine imaging, diagnosis, and therapy simultaneously. As highly‐expressed H_2_S within the disease area as a trigger, multifunctional nanoagents can serve as imaging and therapeutic agents simultaneously. As mentioned previously, the H_2_S functions as an important biological indicator and also has vital roles in a series of physiological functions, such as factors for protecting or killing cells. However, the application of most H_2_S donors is restricted by the short half‐life and low hydrophilic property. Due to these limitations, several H_2_S‐releasing nanomaterials were developed for various disease therapies. In this following section, these latest nanoagents designed for tumor diagnosis and treatment enabled by endogenous H_2_S activation will be discussed (**Table** **4**). Additionally, the exogenous H_2_S delivering nanoplatforms employed for tumor therapy, ischemic/reperfusion protection, and transplanted organ preservation will be summarized.

**Table 4:** Multifunctional nanoplatforms for SHTS‐based imaging and therapy

Imaging strategy^a)^	Material	Nanoparticles	Size [nm]	Therapeutic mechanism	Administration	Disease	Ref.
FL	MOF	Cu_2_(ZnTcpp)·H_2_O	120	Photodynamic	Intratumoral	Colorectal cancer	55
NIR	Polymer	Nano‐PT	8.4–15	Photothermal	Subcutaneous	Colorectal cancer	175
PA	Cu	Cu_2_O	21	Photothermal	Intravenous	Colorectal cancer	54
US/MRI	Liposome	AML	≈200	Bubble/H_2_S bomb	Intravenous	Hepatocellula cancer	56
N/A	Silica	DATS‐MSN	≈225 ± 35	GSH triggered‐release H_2_S	Intravenous	Heart I/R injury	176
		DATS‐MSN	≈225 ± 35	GSH triggered‐release H_2_S	Intravenous	Myocardial I/R Injury	57
		DATS‐MSN	175 ± 35	GSH triggered‐release H_2_S	Preoperative treatment	CAV	177

^a)^ I/R: Ischemic/reperfusion; US: Ultrasound; MRI: Magnetic resonance imaging; CAV: Cardiac allograft vasculopathy, MSN: Mesoporous silica nanoparticles.

### 5.1 Endogenous H_2_S‐Triggered Photodynamic Therapy

**Figure 15:**
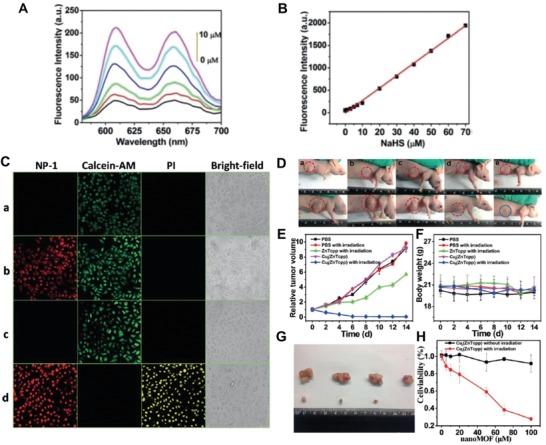
A) The change of fluorescence spectra of NP‐1 after incubating with HS^−^ (0 × 10^−6^ to 10 × 10^−6^ m). B) The linear relationship between the fluorescence intensity of MOF NP‐1 and the NaHS's concentration. C) Confocal images obtained from the HepG2 cells with calcein‐AM and PI staining after the following treatments: a) 10 × 10^−3^ m MOF NP‐1; b) 10 × 10^−3^ m MOF NP‐1 + 50 × 10^−3^ m NaHS; c) 10 × 10^−3^ m MOF NP‐1 + irradiation; d) 10 × 10^−3^ m MOF NP‐1 + 50 × 10^−3^ m + irradiation. D) Optical images of the nude mice bearing HCT‐116 tumor with different treatments (before treatment, upper raw; after treatment, down raw): a) PBS administration; b) PBS injection followed by irradiation; c) ZnTcpp administration followed by irradiation; d) Cu(ZnTcpp) (MOF NP‐1) injection; e) Cu(ZnTcpp) injection followed by irradiation. E) The growth inhibition curve of tumor among different treatment groups. F) The body weight of mice from different therapy. G) Optical images of tumors extracted from MOF NP‐1 (upper row) and control (down raw) groups after irradiation. H) The MTT assay of the HCT116 cells treated with various concentrations of MOF NP‐1 with or without irradiation. Reproduced with permission.^55^ Copyright 2016, Wiley.

Under a specific wavelength (e.g., near‐infrared light), photosensitizing agents generate reactive oxygen species (ROS) for treatment of diseases such as bacterial infection or cancers, referred to as photodynamic therapy (PDT).^178^, ^179^, ^180^ Compared with conventional therapies such as chemotherapy and radiotherapy, PDT is an ideal strategy to treat cancer (i.e., lead the cellular apoptosis and necrosis via the ROS activated) since it is noninvasive, safe, and convenient.^181^ However, photosensitizing agents (e.g., porphyrin) typically cannot elicit an antitumor PDT effect due to their physiochemical features (e.g., hydrophobic) nor are able to diagnose cancer. As such, nanomaterial alternatives have arisen as an attempt to effectively implement this therapeutic strategy. As an example, Ma et al. developed a smart, H_2_S‐triggered MOF nanosensor acted as a photosensitizer after exposure to H_2_S (**Figure** **15**).^55^ This novel MOF, (Cu_2_(ZnTcpp)·H_2_O)*_n_* (NP‐1) was synthesized using a reverse microemulsion system followed by a hydrothermal treatment. NP‐1 reacted quickly with H_2_S within one minute to recover red fluorescence (≈Em610 and Em660). A linear logarithmic relationship was found for the fluorescence intensity and NaHS concentration (from 10–70 × 10^−6^ m) (Figure 15A,B). As a potential photosensitizer, the NP‐1 showed better PDT efficacy than the ZnTCPP precursor (Figure 15C). Specifically, NP‐1 (10 × 10^−6^ m) responded only to laser irradiation (600 nm) to generate ^1^O_2_ when H_2_S (50 × 10^−6^ m) was present. In comparison, when not irradiated or H_2_S was absent, NP‐1 was unable to damage to HepG2 human liver cancer cells (Figure 15C). After intratumoral injection and irradiation, NP‐1 was detrimental to the HCT‐116 cells (high H_2_S levels) and nearly eradicated the entire tumor (Figure 15D–G). Tumor shrinkage was also observed for mice injected with ZnTcpp following irradiation, but the therapeutic effect was relatively poor compared with NP‐1. The role of H_2_S in irradiation‐induced damage was confirmed using HCT‐116 cells (Figure 15H). Although this intelligent nanoplatform, NP‐1 shows significant potential as a H_2_S‐selective photosensitizing agent for PDT of cancer, further functionalization using PEGylation to enable the whole body circulation is highly recommended.

### 5.2 Endogenous H_2_S‐Triggered Photothermal Therapy

**Figure 16:**
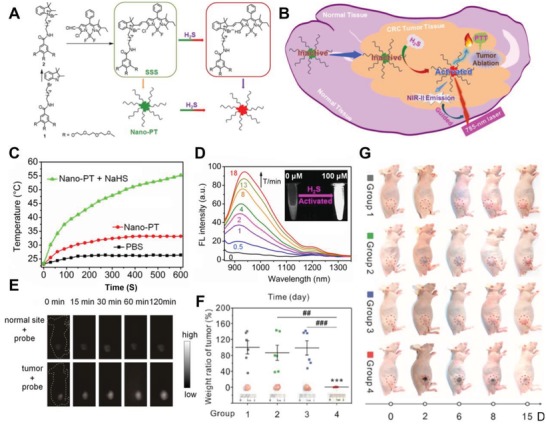
A) Schematic of Nano‐PT synthesis, the chemical structures of the components, and the transformation of SSS after the presence of H_2_S. B) Schematic illustration of the NIR‐II‐guided photothermal therapy for colorectal cancer mediated by Nano‐PT nanoplatform. C) The temperature curves of PBS, Nano‐PT, and Nano‐PT + NaHS (100 × 10^−6^ m) under laser irradiation. D) The change of NIR‐II fluorescence spectra of Nano‐PT during a series of time points (0–15 min) with NaHS (100 × 10^−6^ m), and the NIR‐II image of Nano‐PT after the H_2_S activation. E) The NIR‐II in vivo images of the normal and HCT‐116 tumor tissue on nude mice after on‐site subcutaneous injection of Nano‐PT at different time points. F) The ratios of tumor weight (*W*_d15_/*W*_d0_) among tumors collected from different groups (1) Control; 2) Nano‐PT; 3) Laser; 4) Nano‐PT + Laser) at day 15 and the corresponding photos of representative tumor tissues. G) The optical images of representative mice from different treated groups (1) Control; 2) Nano‐PT; 3) Laser; 4) Nano‐PT + Laser) at a series of time points; tumor sites has been indicated by red circles. Reproduced with permission.^177^ Copyright 2018, Wiley.

As an additional photodynamic treatment, photothermal therapy (PTT) can damage or kill cancer cells by generating vibrational energy in the form of heat after electromagnetic radiation.^37^, ^182^, ^183^, ^184^, ^185^ Many nanomaterials, including gold nanorods and graphene, have been employed as PTT photosensitizers using NIR excitation.^157^, ^186^ Nevertheless, the scatted nanoagent would cause further damage to surrounding normal tissues after a laser applied. Therefore, targeting or selective ability is strongly required. Recently, an innovative nanoagent (Nano‐PT) was synthesized via self‐assembly of a H_2_S activated small molecule that is consist of a hydrophilic tail and a BODIPY core (**Figure** **16**A,B).^175^ As previously mentioned, the absorption wavelength of BODIPY changed after interaction with H_2_S. The variation (i.e., the change of wavelength from Ab540 to Ab790) enables the Nano‐PT to absorb NIR irradiation (785 nm laser, 5.37 W cm^−2^) and produce heat that can reach around 55° after 10 min of irradiation (Figure 16C). However, the temperature of the Nano‐PT solution only slightly increased when H_2_S was absent. After the introduction of H_2_S, a bright NIR‐II fluorescence signal (around Em950) was activated, and continuingly enhanced in a time‐dependent pattern, with a LOD value at 106 × 10^−9^ m (Figure 16D). With such effective sensitivity, the HCT‐116 tumor could be identified from normal tissue 2 h post the subcutaneous injection of Nano‐PT (Figure 16E). Importantly, a 20‐degree temperature difference between normal (41.8 °C) and tumor (60.9 °C) tissue could well prevent accidental injury of nearby tissue. Furthermore, PTT mediated by Nano‐PT successfully ablated the HCT‐116 tumor and limited any noticeable damage to the surrounding healthy tissue (Figure 16F,G).

**Figure 17:**
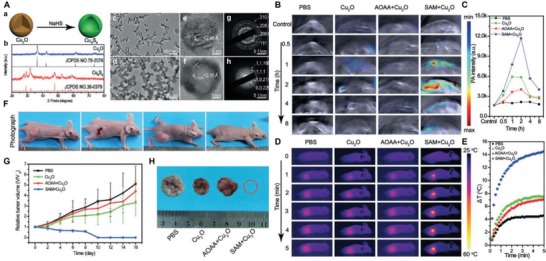
A) The characterization of Cu_2_O and Cu_9_S_8_ nanoparticles: a) Schematic of the H_2_S‐induced transformation of Cu_2_O to Cu_9_S_8_; b) XRD patterns of the Cu_2_O and Cu_9_S_8_ nanoparticles; c,d) TEM images of the Cu_2_O and Cu_9_S_8_ nanoparticles; e,f) HR‐TEM images of the Cu_2_O and Cu_9_S_8_ nanoparticles; g,h) SAED patterns of the Cu_2_O and Cu_9_S_8_ nanoparticles. B) In vivo PA images of the mice bearing HCT‐116 tumor at various time points with different treatments. C) The corresponding PA intensities within the tumors. D) The in vivo thermal imaging of the mice carrying HCT‐116 tumor through a period of time after different treatments. E) The corresponding temperature change curve post 5 min irradiation. F) The optical images of representative mice bearing tumor from various therapeutic groups at day 16 posttreatment. G) The growth curve of tumor from different groups from 0 to 16 days. H) The representative tumor tissue harvested at day 16 postdifferent treatments. Reproduced with permission.^54^ Copyright 2018, Wiley.

By intratumorally or subcutaneously administration, these nanoplatforms are able to treat noticeable tumors with PDT and PTT. However, these strategies are limited for clinical applications that often require simultaneous diagnosis and therapy. To achieve this, Yang's lab recently designed a H_2_S activated nanomaterial, Cu_2_O (21 nm), for colon cancer (HCT‐116, CBS overexpression) theranostics (**Figure** **17**A).^54^ After encountering endogenous H_2_S at the tumor site, Cu_2_O formed Cu_9_S_8_ which absorbed NIR irradiation (808 nm) and increased the tumor tissue temperature by 20.7 °C. Additionally, the formation of Cu_9_S_8_ provided a stable PA imaging agent that was unaffected by pH variations or GSH. For better efficiency, SAM (S‐adenosyl‐l‐methionine) or AOAA (amino‐oxyacetic acid) were administered by intravenous injection as a CBS activator and inhibitor, respectively. After supplementation of Cu_2_O with SAM, increased PA intensity was found at the tumor site (Figure 17B,C). While PA signal from Cu_2_O was detected, it failed to identify the tumor area due to its relatively lower intensity. Similarly, the CBS activator dramatically enhanced the temperature elevation with SAM + Cu_2_O treatment (15 °C), which was twice that of the Cu_2_O treated mice (Figure 17D,E). After two weeks of treatment with SAM + Cu_2_O and laser irradiation, HCT‐116 tumor‐bearing mice were completely eradicated (Figure 17F–H). In comparison, the size of the tumor treated with Cu_2_O + irradiation only slightly decreased. Thus, the reported Cu_2_O nanoparticle was an intelligent theranostic agent for clinic application after supplementation with SAM.

### 5.3 Nanoplatforms as Exogenous H_2_S Delivery System

**Figure 18:**
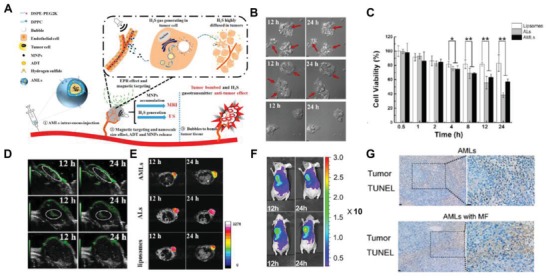
A) Schematic of the combination tumor therapy mediated by the AMLs (anethole dithiolethione (ADT)‐loaded magnetic nanoliposome) nanoplatform. B) The optical images of the cellular morphology change and the bubble generated inside at 12 and 24 h after the incubation with AMLs (upper raw), ALs (middle raw), and liposomes (down raw) respectively; the generated bubbles and the serious membrane disruption have been indicated by red arrows. C) The HepG2 cell viability after the incubations with various samples for different time periods; the statistical difference is shown by **p < 0.01 and *p < 0.05. D) The in vivo ultrasonic and E) T2 MR imaging of the HepG2 tumor area at 12 and 24 h post the injections of AMLs (upper raw), ALs (middle raw), and liposomes (down raw). F) The DiR‐fluorescence images of a HepG2 bearing mice with the injection of DiR‐AMLs under external magnetic field (down raw) or no (upper raw) at 12 and 24 h. G) The TUNEL assay on tumor tissue obtained. Scale bars = 20 µm. Reproduced with permission.^56^ Copyright 2017, American Chemical Society.

Low concentrations of H_2_S are widely known to aid the proliferation of cancer cells and surrounding vessels.^4^, ^61^ However, sufficient H_2_S quickly released in tumor tissue affects cellular metabolism and has a toxic effect on tumor cells.^4^ Exploiting this, Liu et al designed a H_2_S‐generating “nanobomber” for cancer therapy (**Figure** **18**A).^56^ This nanoliposomes (AML) was loaded with the H_2_S donors, anethole dithiolethione (ADT) and magnetic nanoparticles (MNPs), and had a diameter around 200 nm. The ADT could be activated enzymatically to continuingly release significant H_2_S gas, eventually forming microsized bubbles (Figure 18B). The H_2_S bubbles rapidly occupied most of the intracellular space and caused the apparent morphology changes,) which was strongly cytotoxic to HepG2 cells, with more than 40% death after 12 h (Figure 18C). These microbubbles were detected using ultrasonic imaging. After loading with MNPs, the AML accumulated in the tumor area under a magnetic field, which was around 3.4 times of that of Als (without MNPs) at 4 h postinjection (Figure 18D–F). Ultrasonic treatment was then applied was applied to burst the intratumoral microsized bubbles and subsequently induce physical damage and H_2_S‐induced cytotoxicity to the tumor tissue. The magnetic‐guided as therapy successfully induced cell apoptosis (with 21.5 ± 7.4%) and suppressed the tumor growth up to 7 days. However, treatment without the magnetic field showed relatively lower therapeutic effect and decreased apoptosis rates (15.4 ± 4.5%) (Figure 18G). In conclusion, this combined imaging system strongly enhanced the targeting accuracy during the treatment w will also providing the “H_2_S air bomber” for a novel cancer therapy strategy.

**Figure 19:**
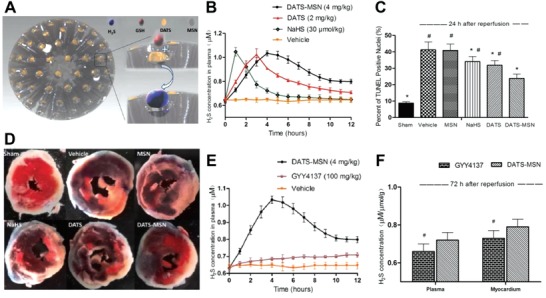
A) Schematic of a GSH‐mediated DATS‐MSN nanoplatform for sustained release of H_2_S. B) The curve of H_2_S concentrations in the mice plasma from different groups overtimes. C) The apoptosis rate of cardiomyocytes after the myocardial ischemia/reperfusion (I/R) injury, which is shown by positive cell percentage in the TUNEL staining. D) The representative photographs of mid‐myocardial cross sections with TTC staining at 72 h post I/R injury. E) The concentration of H_2_S within the plasma from GYY4137 or DATS‐MSN treated groups during the 12 h after I/R injury. F) H_2_S concentrations in the plasma and myocardium from GYY4137 or DATS‐MSN treated mice at 72 h after I/R injury. Reproduced with permission.^57^ Copyright 2017, Nature Research.

Supplementation of H_2_S can help preserve organs and protect injuries triggered by ischemia/reperfusion by various antiapoptotic, antiinflammatory and antioxidative methods.^187^, ^188^, ^189^ However, most H_2_S donors cannot produce decent protection due to burst release and poor solubility, such as the NaHS or diallyl sulfide (DATS). Mesoporous silica nanoparticles (MSNs) have arisen as ideal nanoplatforms due to their large surface area that can be diversely functionalized, adjustable pore size for loading various cargo (e.g., the hydrophobic drug), and overall biocompatibility.^190^, ^191^ Recently, Wang's lab successfully developed DATS‐loaded MSNs as a H_2_S‐generating platform for protecting organs from I/R injury and transplantation.^57^, ^176^, ^177^ These MSN (175–225 nm) efficiently carried DATS at the surface pore (≈2 nm) because of the high affinity between DATS and Si‐OH, with an entrapment rate around 99% (**Figure** **19**A).^176^ A sustained DATS release profile (reaching about 80 min) was achieved after loaded on MSN and in the presence of GSH in the solution. In turn, the amount of H_2_S released from DATS alone quickly declined after only one hour. The supplementation of DATS‐MSN in the preserving solution effectively reduced inflammation in the transplanted organ by downregulating the expression level of intercellular adhesion molecule‐1 (ICAM‐1) and vascular cell adhesion molecule‐1 (VACM‐1).^177^ Notably, the DATS‐MSN continuingly released H_2_S into the plasma for up to 12 h, while NaHS and DATS quickly decreased after one or three hours respectively (Figure 19B).^57^ The administration of DATS‐MSN reduced myocardial apoptosis by approximately 15% at 24 h post‐reperfusion. Additionally, DATS‐MSN and substantially decreased I/R injury in myocardial tissues, which was confirmed using TTC staining (percentage of infarction area (INF)/area at risk (AAR)) (Figure 19C,D). More importantly, the DATS‐MSN exhibited superior protection of the heart after I/R injury in comparison to GYY4137, a conventional H_2_S donor with slow release kinetics (Figure 19E,F).

## 6 Conclusion and Future Outlook

Undoubtedly, early diagnosis significantly contributes to attaining successful therapeutic interventions.^192^ Early diagnosis—especially for cancer—is likely to increase the efficacy of nearly every therapy, ranging from surgery, chemotherapy, radiotherapy to immunotherapy. In addition screening specific diseases' biomarkers (e.g., tumor surface markers), proper surveillance of the influential gasotransmitters would effectively aid disease diagnosis at early stages.^193^ Of these, H_2_S is vitally important in a series of signaling pathways associated with various physiological (e.g., antiinflammation and antiapoptosis) and pathological effects (e.g., tumor progress, etc.).^194^ Additionally, the high toxicity of H_2_S further emphasizes the importance of monitoring H_2_S, especially for potential air exposures. Currently, several organic probes have been implemented for detecting/imaging H_2_S. However, widespread applicability is restricted by their poor physiochemical conditions, including relatively weak sensitivity and limited circulation.^13^, ^14^

**Figure 20:**
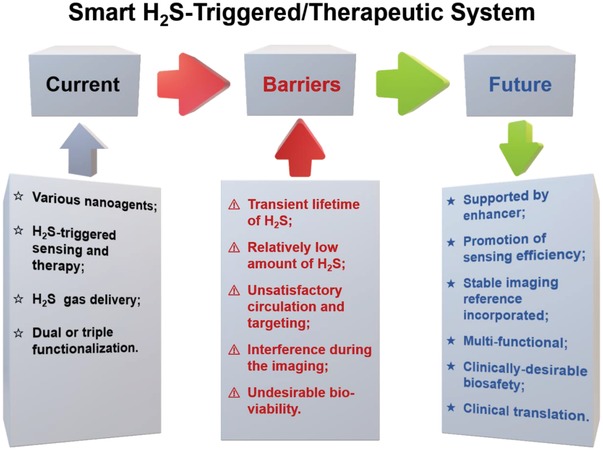
The current status and barriers that need to be overcome for future development of smart H_2_S‐triggered/therapeutic system (SHTS).

Advanced nanomaterials have demonstrated desirable properties as multifunctional platforms for imaging and therapy.^49^, ^195^ In recent years, nanomaterials have been continually developed as novel probes for H_2_S‐triggered detection, imaging, and therapy (Figure 1). This review summarizes and discusses all SHTS‐based nanomedicines to date, focusing on H_2_S imaging of cancer cells and in tumor‐bearing mice as well as for disease therapy (e.g., cancer or I/R injury) (**Table** **5**). More specifically, various H_2_S imaging approaches using fluorescence, LSPR, UCL, NIR, PA, and PET modalities are summarized. Therapeutic strategies, such as photodynamic and photothermal therapy, influenced by the presences of H_2_S are also discussed in detail. To provide more ideas for the H_2_S related treatments, the H_2_S generated nanoplatforms have been included as well. Undeniably, the development of SHTS‐based nanomedicine has seen much progress accelerated by the efforts of researchers. However, there are still several principles and challenges that need to be addressed in future H_2_S‐nanoprobe designs. Below we provide a series of considerations regarding these crucial issues for future SHTS‐base nanomedicine innovation and translation (**Figure** **20**).

**Table 5:** The nanoagents involved in SHTS

Material^a)^	In vitro detection/imaging				In vivo imaging	Therapy		Number of Application		Features	
	CM	EC	FL	Others		H_2_S‐triggered	H_2_S delivery	In Vitro	In Vivo	Cons	Pros
NMNCs/NPs	✓✓		✓	DFI				19	0	Limited imaging depth; In vitro detection only; Expensive	High sensitivity; Direct reaction with H_2_S; Eye‐visible detection; Reusability
C‐dot		✓	✓					7	0	Limited imaging depth; In vitro detection only	High biocompatibility; Affordable and reproducible preparation
Cu		✓✓	✓		PET	PT		7	2	Low biocompatibility	Quick and direct reaction with H_2_S; Gas detection
Silica			✓		NIR/PA		✓	1	5	Relatively high cytotoxicity; Limited circulation	Easy functionalization; Strong capability in drug loading
LNPs/PMNs			✓		NIR/US/MRI	PT/BB		0	3	Relatively low stability	Desirable biocompatibility and circulation
MOF			✓		FL	PD		8	1	Limited circulation; Relatively large size; Poor in vivo imaging (FL)	Large surface area for modification; Direct reaction with H_2_S via the cation carried
UCNPs				UCL	UCL			2	3	Relatively high cytotoxicity	Desirable imaging penetration; Consistent imaging reference for calibration

^a)^ NMNCs/NPs: Noble metal nanoclusters/nanoparticles; CM: Colorimetry; EC: Electrochemistry; FL: Fluorescence; DFI: Dark field imaging; UCL: Upconversion luminescence; NIR: Near infrared; PA: photoacoustic imaging; PET: positron emission tomography; PD: Photodynamic; PT: Photothermal; BB: Bubble bomb.

### 6.1 Challenge

Due to physiochemical properties, H_2_S quickly dissolves in water and results in the formation of HS^−^ and S^2−^ that introduce interference. Additionally, toxic H_2_S generated from cells is processed rapidly by anabolism and catabolism. Due to this dynamic nature, real‐time imaging of H_2_S is highly demanded to inform the location/status of disease (e.g., cancer) following therapy. In summary, a specific, sensitive, and multifunctional H_2_S sensor with excellent circulation (for reaching the specific area) is ideal for H_2_S detection and therapy.

### 6.2 Influence of Size, Shape, and Charge

The morphology of nanomaterials, especially size, directly affects the optical features (e.g., LSPR) and contacting area. Both of these aspects are strongly related to the sensitivity toward H_2_S. Additionally, large nanoparticle (>200 nm) tend to absorb more serum proteins (34% absorbance) compared with smaller ones (80 nm, with 6% absorbance). This results in only smaller nanoparticles having a circulation half‐life suitable for imaging.^196^ Additionally, the nanomaterials biodistribution is significantly affected by their shape and surface charge.^197^, ^198^, ^199^ For instance, tumor tissue accumulation is enhanced with negatively charged NPs.^198^, ^199^ Thus, varying the diameter, shape, and charge alter biodistribution and tumor penetration and subsequently influence the efficiency of imaging and therapy.^200^, ^201^

### 6.3 Surface Modification

Although the H_2_S detection (e.g., solution, serum or H_2_S in the air) can be performed with unmodified nanomaterials, surface modifications (e.g., PEGlaytion, acetylation, amino acid or ligand/antibody functionalization) greatly increase their stability, biocompatibility, circulation and targeting for in vivo sensing/delivery.^202^, ^203^ Other surface modifications of functional groups or material (e.g., Cyclam‐Cu^2+^ or FRET acceptor)^45^, ^122^ can impart an alternative strategy that affords a specific nanomaterial (such as Au nanorod with photothermal strategy) with H_2_S‐selectivity.

### 6.4 Accuracy of Real‐Time H_2_S Concentration

During in vivo imaging, interfering background signal from tissue autofluorescence (e.g., skin) greatly affects H_2_S visualization. Although most in vivo NIR or PET imaging agents limit the autofluorescence background, the accuracy of H_2_S detection or imaging would be further influenced by the variation among individuals. As an ideal imaging system, UCNPs can greatly reduce autofluorescence. Additionally, the unique ratiometric strategy applied (i.e., the ratio of specific emission/a control emission) ensure sensing accuracy. Thus, we believe the incorporation of a reference emission using surface modification or reagent loading will increase imaging accuracy during diagnosis and therapy.

### 6.5 Sensitivity Enhancement for In Vivo Imaging

As mentioned above, the biological half‐life of H_2_S is short. Typically, biological concentrations are generally lower than the LOD of most nanoagents. To improve the detection performance, an enhancement (*e.g*, SAM) agent is strongly recommended, especially for H_2_S‐triggered therapeutic nanoplatforms.^54^

### 6.6 Therapeutic Strategy

A series of combined therapies including photodynamic, photothermal, and gas‐generated treatments, have been listed in this review. These smart nanoplatforms are all H_2_S‐regulated and mitigate damage to surrounding tissue. However, the potential problems, including the releasing speed and the concentration of H_2_S generated within a certain area, must be controlled. Meanwhile, additional agents, such as chemical drugs or vaccine adjuvants (e.g., CpG ODN) could be further loaded for combined chemotherapy or immunotherapy after H_2_S activation.

### 6.7 Applications and Selection of Nanosensor

Given diverse applications for SHTS‐based nanomedicine, proper nanoplatform selection is critical. For the detection of H_2_S in solution, biosample, and air, the priority of nanosensor selection is the selective, sensitivity, and practicality. For instance, the sensors with a physical supporting (e.g., supporting membrane) or an eye‐visible colorimetric examination would be more practical and convenient. Alternatively, biocompatibility and circulation half‐life are the key factors for in vivo imaging and therapy. Although great progress has been made in the development of nanomaterials as H_2_S sensors with high sensitivity and selectivity, only a few can apply in the in vivo assay due to the bad biocompatibility and circulation. Thus, to promote the real application of SHTS based nanomedicine and its following clinic translation, more efforts should be dedicated to investigating these aspects.

In a sharp comparison of general strategies, the advances of nanotechnology enable us to combine various functions into one nanoagent. With SHTS‐based nanomedicine, we are able to detect and imaging H_2_S for different applications, and also induce specific therapy following the diagnosis. The increasing interest in real‐time H_2_S imaging and high performance of SHTS would encourage the further investigation of the following translation in the clinic, which will greatly improve the diagnosis of various H_2_S diagnosis and benefit the patients via a safe and efficient therapeutic strategy.

## Conflict of Interest

The authors declare no conflict of interest.
